# A new DNA extraction method (HV-CTAB-PCI) for amplification of nuclear markers from open ocean-retrieved faeces of an herbivorous marine mammal, the dugong

**DOI:** 10.1371/journal.pone.0278792

**Published:** 2023-06-07

**Authors:** Vicky Ooi, Lee McMichael, Margaret E. Hunter, Aristide Takoukam Kamla, Janet M. Lanyon

**Affiliations:** 1 School of Biological Sciences, The University of Queensland, St Lucia, Queensland, Australia; 2 School of Veterinary Science, The University of Queensland, Gatton, Queensland, Australia; 3 U.S. Geological Survey, Wetland and Aquatic Research Center, Sirenia Project, Gainesville, Florida, United States of America; 4 Aquatic Animal Health Program, College of Veterinary Medicine, University of Florida, Gainesville, Florida, United States of America; 5 African Marine Mammal Conservation Organization, Dizangue, Littoral, Cameroon; Universidade Lisboa, Instituto superior Técnico, PORTUGAL

## Abstract

Non-invasively collected faecal samples are an alternative source of DNA to tissue samples, that may be used in genetic studies of wildlife when direct sampling of animals is difficult. Although several faecal DNA extraction methods exist, their efficacy varies between species. Previous attempts to amplify mitochondrial DNA (mtDNA) markers from faeces of wild dugongs (*Dugong dugon*) have met with limited success and nuclear markers (microsatellites) have been unsuccessful. This study aimed to establish a tool for sampling both mtDNA and nuclear DNA (nDNA) from dugong faeces by modifying approaches used in studies of other large herbivores. First, a streamlined, cost-effective DNA extraction method that enabled the amplification of both mitochondrial and nuclear markers from large quantities of dugong faeces was developed. Faecal DNA extracted using a new ‘High Volume- Cetyltrimethyl Ammonium Bromide- Phenol-Chloroform-Isoamyl Alcohol’ (HV-CTAB-PCI) method was found to achieve comparable amplification results to extraction of DNA from dugong skin. As most prevailing practices advocate sampling from the outer surface of a stool to maximise capture of sloughed intestinal cells, this study compared amplification success of mtDNA between the outer and inner layers of faeces, but no difference in amplification was found. Assessment of the impacts of faecal age or degradation on extraction, however, demonstrated that fresher faeces with shorter duration of environmental (seawater) exposure amplified both markers better than eroded scats. Using the HV-CTAB-PCI method, nuclear markers were successfully amplified for the first time from dugong faeces. The successful amplification of single nucleotide polymorphism (SNP) markers represents a proof-of-concept showing that DNA from dugong faeces can potentially be utilised in population genetic studies. This novel DNA extraction protocol offers a new tool that will facilitate genetic studies of dugongs and other large and cryptic marine herbivores in remote locations.

## Introduction

Genetic variation between individuals frequently influences the evolutionary resilience of species [1, 2]. Small populations lacking in gene flow have low genetic diversity, which may restrict their ability to adapt to environmental changes, leaving them vulnerable to extinction [3]. As threatened wildlife species dwindle in number, studies addressing genetic variation within and between populations become crucial for continued conservation efforts [4]. To obtain critical genetic information on free-ranging wildlife, most studies rely on direct sampling of body tissues (e.g., blood, skin) as a source of high quality DNA [5]. However, there may be logistical and/or ethical challenges in direct sampling of large, rare, cryptic, or elusive species [6]. In contrast, non-invasive sampling through collection of animal traces, such as faeces, shed hairs, or sloughed skin as a source of DNA, mitigates the need to invasively capture, restrain or in some cases, directly observe the target wildlife [7]. One drawback of sampling biological traces is that quantity and quality of DNA may be compromised [7]. However, if collected and processed appropriately, non-invasive samples may provide comparable genetic information as obtained through direct sampling, e.g., [8].

Utilisation of faeces as an alternative source of DNA has its challenges. Apart from a generally low yield of amplifiable target DNA, faeces may contain polymerase chain reaction (PCR) inhibitors that originate from digestive contents or the environment in which they are voided [9]. DNA from herbivore faeces is often more difficult to amplify compared to that of carnivores, since these may contain plant secondary metabolites that inhibit PCR [10]. The use of faecal DNA for genetic studies of marine species is even more restricted due to degradation and contamination in seawater [11]. Furthermore, the age of faecal samples may impact retrieval of target DNA, i.e., higher yield and quality of target DNA may be extracted from fresh compared to old scats [12].

The bulk of mammalian faeces comprises water and soluble molecules such as mucin, and the remainder consists of undigested food and microflora [13]. A small portion of faeces is composed of epithelial cells sloughed from the host’s gastrointestinal tract [14]. Based on the assumption that sloughed cells would adhere to the surface of the egesta as it passes through the gastrointestinal tract, some sampling protocols swab the surface of scats to obtain these cells [15], whilst others resort to either homogenising the faecal sample or scraping, peeling, and/or washing its surface [9]. Surprisingly, the distribution of target cells and thus host DNA within faeces appears to have remained underexplored despite increasing efforts to isolate DNA from scats. Although various extraction methods have been used to retrieve host DNA from faecal samples, there is no ‘one-size-fits-all’ approach that works optimally for all species. Depending on the application, a more affordable DNA extraction method, if available, may also be more practical when a large number of samples needs to be processed. Amongst the different DNA extraction techniques available, some of the most widely used include the QIAamp Fast DNA Stool Mini Kit (hereafter referred to as QIAamp) [8, 16], phenol-chloroform-isoamyl alcohol (PCI) [12, 17], and cetyltrimethyl ammonium bromide (CTAB) [18]. The 2CTAB/PCI protocol, a combination of the latter two procedures, is a particularly notable extraction technique as it has demonstrated success in extracting DNA from herbivore faeces [19].

Dugongs (*Dugong dugon*) are vulnerable marine mammals that are declining throughout their range from East Africa to the south Pacific islands of Vanuatu [20]. Dugongs are challenging to study as they often reside in inshore, turbid waters and remote locations where access may be limited [21]. Dugongs spend most of their lives underwater, and their short surface intervals [22] make them difficult to approach and sample directly. Consequently, few genetic data are available for dugongs throughout much of their range. Of all regions supporting dugongs, northern Australia has been best studied [23] due to relative accessibility of some locations by researchers and intermittent recovery of carcasses. Conversely, in many regions outside Australia, dugong densities are low and variable; some populations have been extirpated, some are functionally extinct [24], and most are in decline [20]. These challenges to studying dugongs has rendered a non-invasive DNA sampling approach, i.e., via faeces, increasingly desirable as it may enable population genetic studies in areas where direct sampling is impracticable.

Early genetic studies of dugongs analysed mtDNA from recovered carcasses [25, 26]. Maternally-inherited mtDNA has high mutation rates, making it an effective marker for distinguishing deep taxonomic relationships and broad population structure [27]. Although mitochondrial studies suggested two dugong maternal lineages in Australia (i.e., north-western, and eastern populations), separated at Torres Strait [26], it was only when nuclear microsatellites were used that finer scale population structuring was found within southern Queensland [28]. A more recent study evaluated population structure of dugongs along the entire eastern Queensland coast, using the same set of microsatellites, and found an abrupt genetic break at the Whitsundays region, effectively separating northern and southern clusters [29]. Two less distinct subclusters were found within each of these main clusters, but a lack of tissue samples along the more remote northern coast limited discernment of population structuring along the entire coast. These patterns were also supported by analysis using 10,690 single nucleotide polymorphism (SNP) loci [29]. A SNP represents variation of a single nucleotide base within the genome of different individuals, where the least abundant allele or nucleotide occurs in more than 1% of the population [30]. Although a greater number of SNPs are required to provide the same resolution as microsatellites due to the biallelic nature of SNPs as opposed to the multiallelic nature of microsatellites, fewer genotyping errors associated with SNPs, and their abundance throughout the genome have led to an increase in their use [31]. More importantly, the small target regions for SNP amplifications would allow for higher PCR amplification success in more degraded DNA such as those from faeces [32].

In several areas where dugong tissues are difficult to obtain, faecal samples have been collected, although their use remains limited as only mtDNA has been successfully amplified, despite tests on nuclear markers [26]. Acquiring successful amplification of nuclear markers is more challenging because target DNA from faeces is often highly degraded, and the amount of available nDNA is less than that of mtDNA, due to the nature of a diploid cell containing many mitochondria but only two copies of the nuclear genome [33]. That said, a recent study has successfully amplified both nuclear and mitochondrial markers from faeces of the African manatee (*Trichechus senegalensis*) and Florida manatee (*Trichechus manatus latirostris*), using the QIAamp method with some modifications, and an additional purification step to remove PCR inhibitors [34]. Manatees, phylogenetically related to dugongs, are generalist herbivores that consume highly fibrous and abrasive macrophytes [35], whereas dugongs are seagrass specialists that preferentially graze on seagrasses with high nitrogen and low fibre content [36, 37]. As dugongs have high apparent digestibility of low fibre seagrass [38], they produce less fibrous faeces compared to manatees [34]. Although these sirenians possess some differences in terms of diet and digestive function [39], it is theoretically likely that the extraction methods used for manatees [34] may work in dugongs since their less fibrous diets may reduce the occurrence of PCR inhibitors in their stools.

This study sought to establish a robust and cost-effective faecal DNA extraction protocol and enhance faecal sampling methodologies to enable successful amplification of nuclear and mitochondrial markers used in population genetics studies of dugongs. The specific objectives of this study were threefold: (1) To develop and validate a streamlined and cost-effective faecal DNA extraction method that can retrieve sufficient yield of dugong DNA, enabling the consistent amplifications of nuclear and mitochondrial markers from dugong faeces. Zinc Finger X-chromosomal protein encoding ZFX gene and dugong-specific control region of mtDNA were the nuclear and mitochondrial markers used, respectively. (2) To investigate whether the amount of target DNA differed between the outer versus inner layers of faeces with different levels of environmental exposure and determine if the amplification success of both markers differed between the faecal groups. Based on the prevailing assumptions, we hypothesised that the outer surface layers of a dugong stool would contain more dugong DNA, and that fresher faeces would achieve higher amplification success of those markers. (3) To show a proof-of-concept that nDNA extracted from dugong faeces could be used in their genetic population studies through the amplification of ten previously developed SNPs [29], from fresh dugong faeces, using the optimal DNA extraction protocol. A representative SNP was also amplified in dugong faeces of different environmental exposure levels (EELs) to explore the impacts of degradation on SNP amplification success.

## Methods

### Field sampling

Two types of dugong faeces were collected: *ex-ōceanum* faeces were those that were collected when dugongs were held out of water and were thus uncontaminated by seawater, whilst *in-ōceanum* faeces were those that were collected from the ocean and were contaminated by seawater.

**Fig 1:**
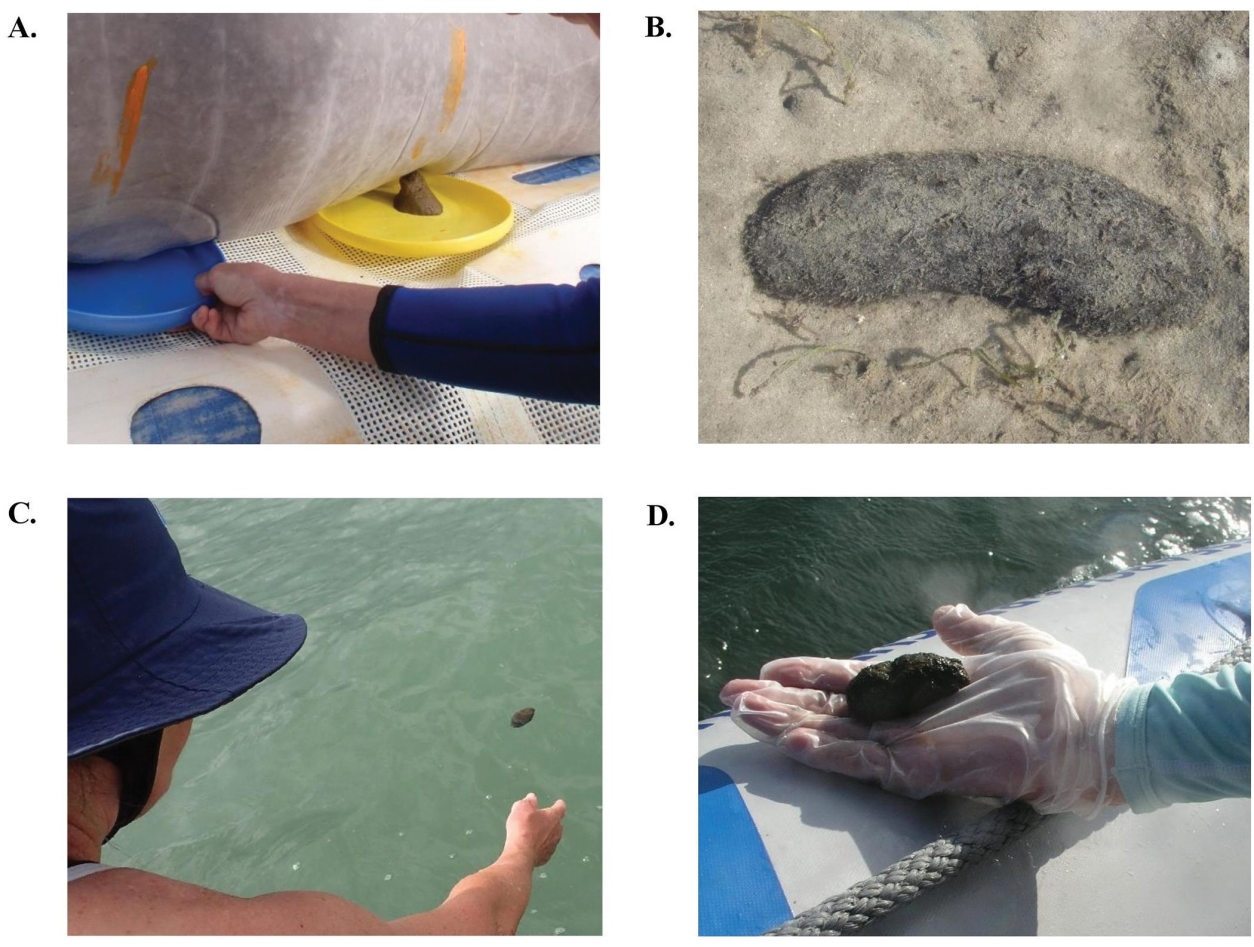
**Photos showing the collection of different dugong faeces.** (A) *Ex-ōceanum* faeces collected onto a Frisbee^®^ from a dugong held on deck. (B) An *in-ōceanum* faeces on the benthos. (C) An *in-ōceanum* faeces floating on the ocean surface. (D) An *in-ōceanum* faeces collected from the ocean surface.

**Fig 2:**
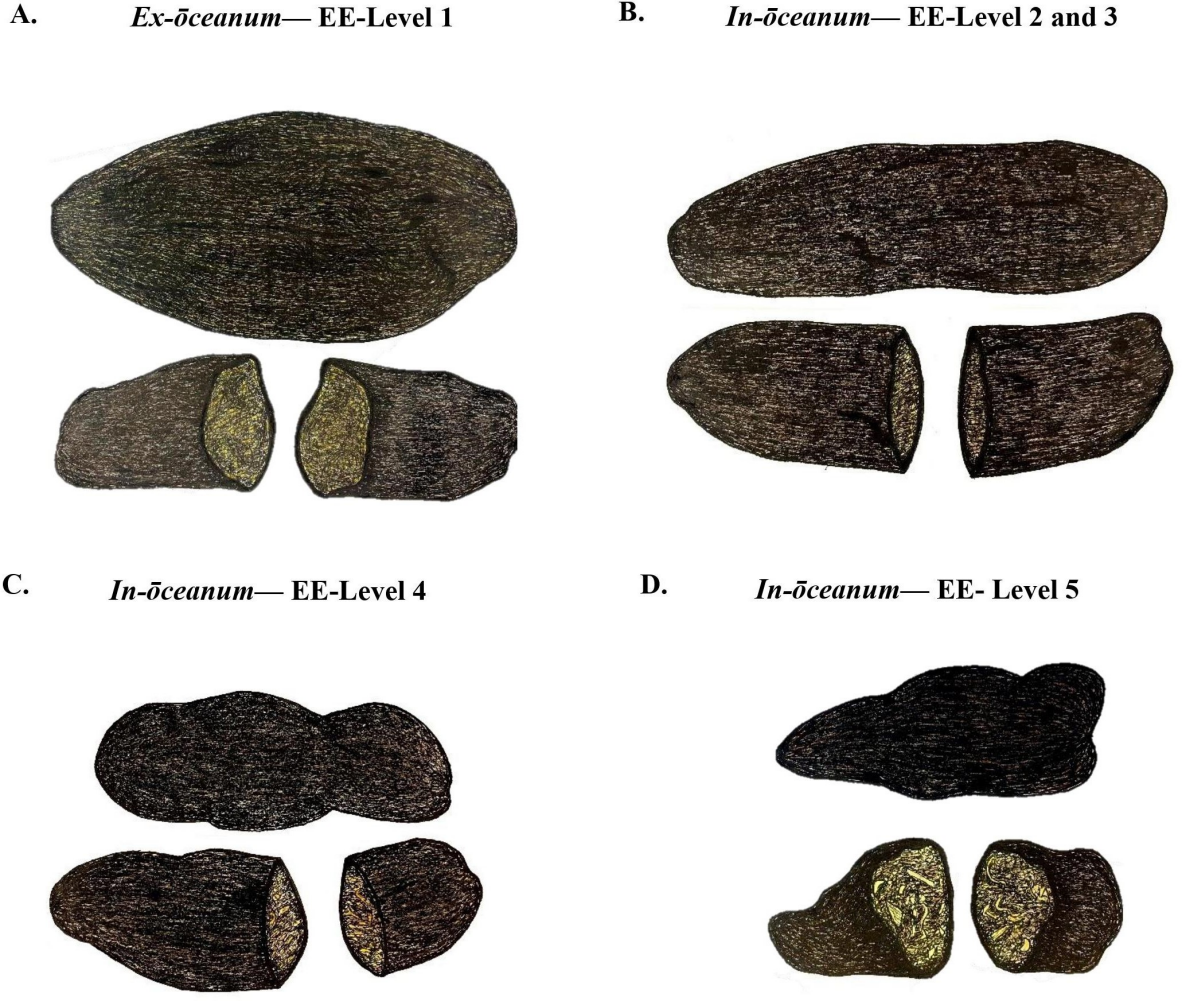
Classification of dugong faeces into five categories as illustrated with an above view (top) and transverse cross-section (bottom). Comparing freshly excreted samples on a boat deck to those most aged in the ocean, a respective transition of surface colour was observed from lighter to darker, yellow-brown, or light brown to black, while sizes trended from large to small, and core composition generally retained higher fibre proportion over time. (A) *Ex-ōceanum*- Environmental Exposure- Level 1 faeces (*ex*-EE-L1), originating beneath the dugong, were flattened in a shallow frisbee. Thus, the above view displays a wider shape (top) and a flatter profile from the side (bottom). (B) *In-ōceanum*- Environmental Exposure- Level 2 and 3 faeces. (C) *In-ōceanum*- Environmental Exposure- Level 4 faeces. (D) *In-ōceanum*- Environmental Exposure- Level 5 faeces.

Matched *ex-ōceanum* faeces (n = 8) and skin tissues (n = 8) were collected from live wild dugongs during the 2018 annual health assessment program in Moreton Bay, Queensland (27.4°S, 153.2°E), Australia [40]. Dorsal skin tissues were sampled using a skin scraper [41], and freshly voided *ex-ōceanum* faeces were collected onto a clean Frisbee^®^ placed under the anus of each dugong held on deck (Fig 1A) [40]. Frozen *ex-ōceanum* faecal samples collected in 2016 (n = 8) and 2017 (n = 8) were also used in this study. *Ex-ōceanum* faeces represent the freshest and least degraded of all faecal types and were categorised under Environmental Exposure Level 1 (*ex-*EE-L1). The surfaces of these faeces were textured, ranging from yellow-brown, or light brown to dark brown, and the inner core was usually more dough-like as opposed to fibrous with yellowish pigmentation (Fig 2A).

*In-ōceanum* faeces of four different EELs or exposure states were collected from the ocean at different time points post-elimination. Fresh *in-ōceanum* faeces with exposure level 2 (*in-*EE-L2) (n = 8) were collected from the benthos (Fig 1B) through free-diving immediately as the feeding herd of dugongs was seen leaving an area (estimated at < 1 h post-elimination). Floating faeces of exposure level 3 (*in-*EE-L3) (n = 8) were collected from the ocean surface (Fig 1C) in an area where the feeding herd was spotted nearby (~ 1–4 h post-elimination, i.e., within a tidal cycle). The *in*-EE-L2 and *in*-EE-L3 faeces are comparable morphologically, i.e., cylindrical (large calibre) of variable length (Fig 2B). Their inner core usually presents comparable to *ex*-EE-L1; however, the proportion of fibre varies. Their colours are usually dark to light brown. Exposure level 4 (*in-*EE-L4) (n = 8) faeces were collected from the ocean surface (Fig 1D) where dugongs were spotted foraging at a distance (~ 5–7 h post-elimination). Morphologically, these stools are usually smaller and/or shorter than *in*-EE-L2 and *in*-EE-L3, though large specimens have been found. The shape of *in*-EE-L4 faeces remains cylindrical, yet jagged edges and lumps are common. Their colours are darker brown to black on the surface and the inner core is more yellowed with a slightly more fibrous nature (Fig 2C). The most eroded faeces, exposure level 5 (*in-*EE-L5) (n = 8), were collected from the ocean surface in an area where no dugongs were spotted (possibly > 7 h post-elimination, i.e., after a full tidal cycle). The *in*-EE-L5 faeces were fragmented into small pieces presumably by ocean currents and/or coprophagous animals since these were exposed to the environment for the longest duration. The most eroded faeces commonly appeared as charred black or dark brown in colour, and the inner core was mostly highly fibrous, though in some cases, these retained less fibre (Fig 2D). As these are recovered in small pieces, the shape can differ from being cylindrical to round (Summary: Table 1).

**Table 1:** Summary on the description and categories of the dugong faecal samples collected and used in this study, including the year and place of collection.

Faecal type	Environmental exposure level	Abbreviation (Faecal Type-Environmental Exposure- Level)	Hours post elimination (*approx*.)	Description of faecal collection	Year of collection	Place of collection
*Ex-ōceanum*	1 (Most fresh)	*Ex*-EE-L1	0	Collected out of water on a boat	2016, 2017, 2018	Moreton Bay, Australia
*In-ōceanum*	2	*In*-EE-L2	~ Less than 1	Collected as feeding herd was seen leaving an area	2022	Fisherman’s Gutter, Moreton Bay, Australia
*In-ōceanum*	3	*In*-EE-L3	~ 1 to 4	Collected where the feeding herd was spotted nearby	2016	Clairview, Australia
*In-ōceanum*	4	*In*-EE-L4	~ 5 to 7	Collected where feeding herd or individual dugongs were spotted at a distance	2015	Cleveland Bay, Australia
*In-ōceanum*	5 (Eroded)	*In*-EE-L5	~ More than 7	Collected in an area where no dugongs were spotted	2016	Newry Island, Australia

Faeces were placed in a resealable plastic bag upon retrieval, stored in an ice-filled portable cooler in the field, and frozen at -20°C at the end of each day. Skin tissues and a single liver tissue sample from a neonate dugong, were used in this study as positive controls; both mtDNA and nDNA have been previously extracted from these tissue types [28].

### Study approach

#### Objective 1: DNA extraction protocol development

To establish an appropriate protocol for DNA extraction from dugong faeces, two faecal sampling and two processing techniques were initially compared, and the approach that recovered the highest amount of total (dugong and exogenous) DNA and target (dugong) mtDNA was used in all further extractions.

QIAamp DNA extraction method was first trialled, but the inability to yield sufficient DNA for consistent amplifications of nuclear (ZFX) marker from dugong faeces using this protocol led to the development of a more robust, streamlined, and cost-efficient new ‘High volume-CTAB-PCI’ (HV-CTAB-PCI) method that isolated DNA from larger volumes of faeces. Total DNA concentration extracted using each protocol, and the amplification success (including each of PCR and triplicate success) of mtDNA control region and ZFX marker were contrasted. PCR success represents the percentage of samples amplified, while triplicate success represents the number of replicates amplified out of three technical replicates for each sample.

#### Objective 2: Faecal layer sampling in faeces of varying quality

The QIAamp method was used to extract DNA from the outer surface and inner core of dugong faeces of four different EELs (all except *in*-EE-L2 faeces, which were yet to be collected at this stage of protocol development). The amount of total DNA used in quantitative PCR (qPCR) reactions was standardised to enable comparisons between faecal samples. Total DNA concentration, relative amount of dugong mtDNA extracted, and amplification success were compared under a two-factorial design. The lack of success with ZFX amplification using this DNA extraction protocol disallowed similar comparisons with the nuclear marker.

The HV-CTAB-PCI method was used to extract DNA from dugong faeces of five different EELs, and both mtDNA control region and ZFX were amplified. At this stage, an additional group of fresh dugong faeces (*in*-EE-L2) was added to further improve chances of achieving successful amplification of ZFX marker from *in-oceanum* faeces. Total extracted DNA concentration, and amplification success of each marker were compared between faeces of different EELs.

#### Objective 3: SNP trial—A proof-of-concept

DNA extracted using the HV-CTAB-PCI method from *in*-EE-L2 faeces was used to trial the amplification of ten SNP primer sets, and amplification success was compared. One SNP marker was selected to be amplified using DNA extracted with the HV-CTAB-PCI method from all *in-ōceanum* faeces, and amplification successes were compared.

### Faecal sampling and processing

**Fig 3:**
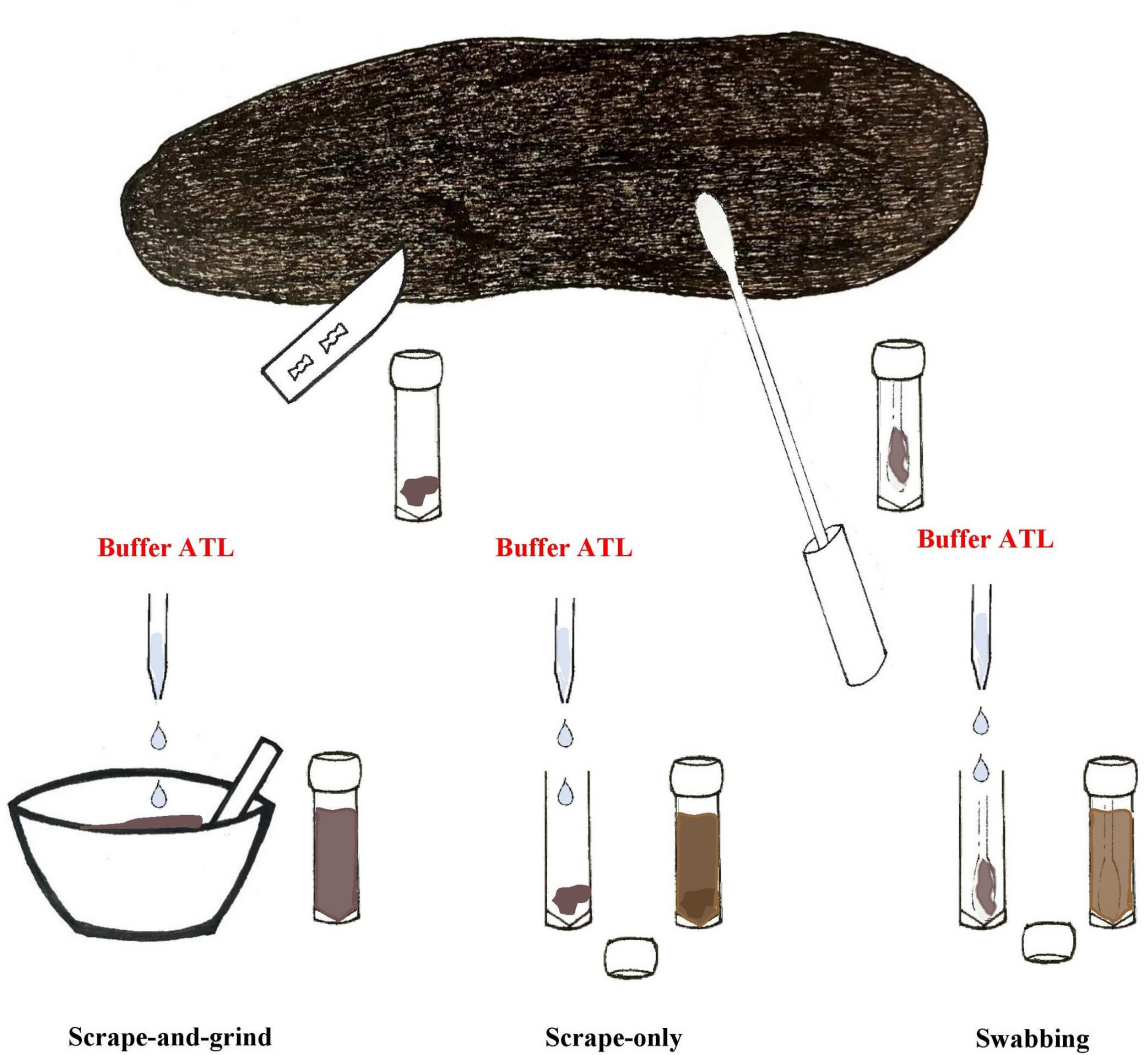
Dugong faecal sampling and processing techniques used in this study. Faecal material was either scraped from the outer surface of faeces or swabbed along the entire faeces for DNA extraction. When faecal material was obtained via scraping, it was either further processed by grinding in liquid nitrogen prior to the addition of lysis buffer (Buffer ATL) or added directly to lysis buffer without any processing. When faecal material was sampled using the swabbing technique, no further processing was performed.

Using a sterile surgical blade, 220 mg of faecal material was scraped from the outer surface of 16 *ex-ōceanum* faecal samples, each weighed and stored in a 2 mL microcentrifuge tube. For inner core sampling, the stool was broken in half and faecal material from the inner core of faeces was sampled. Each faecal sample was then either transferred into a sterilised mortar, and ground into a fine powder with a pestle using liquid nitrogen (N_2_) (referred to as ‘scrape-and-grind’ technique) (n = 8) or left unground (referred to as ‘scrape-only’ technique) (n = 8) (Fig 3).

A further eight *ex-ōceanum* faeces were sampled through swabbing the entire outer surface using a rayon swab (Copan, CA, USA) (referred to as ‘swabbing’ technique). Once the swab tip was entirely covered in faecal material, it was transferred into a 2 mL tube, and the swab’s shaft was trimmed to fit. No further processing was conducted (Fig 3).

### DNA extraction from faeces

#### QIAamp protocol

DNA was extracted using QIAamp Fast DNA Stool Mini Kit (#51604, Qiagen, Germany) from approximately 220 mg of dugong faeces. When swabbing or scrape-only techniques were used, 1 mL of InhibitEX buffer was added into the 2 mL tube containing the faecal sample. When scrape-and-grind technique was used, 500 μL of InhibitEX buffer was added to the mortar with ground faeces for further grinding prior to the transfer of faecal mixture back into the 2 mL tube, and another 500 μL was used to rinse the remaining faeces from the mortar into the same tube. The resultant mixture was vortexed for 1 min (Note: swab was removed after vortexing) and centrifuged for 1 min at 20,000 *g* (14,000 rpm) to pellet the stool particles. The remainder of the extraction was performed following manufacturer’s instructions, but by using 800 μL of supernatant from the lysate, and subsequently equal amount of Buffer AL and 99.9% ethanol, with a final 100 μL elution volume. A step-by-step version of this protocol can be found in the protocol.io repository [42].

#### HV-CTAB-PCI protocol

An extraction protocol was developed using lysis buffers and general concept of the 2CTAB/PCI method [19]. Approximately 1 g of faecal material was processed using the scrape-and-grind technique, and 1 mL of Lysis Buffer 1 (LB1: CTAB 2%, Tris–HCL 100 mM, EDTA 20 mM, NaCl 1.4 M, pH 7.5) was added to the mortar to further grind the powdered faeces before the mixture was transferred into a 15 mL tube. This was repeated twice to ensure that any remaining faecal material in the mortar was collected. Another 2 mL of LB1 was added into the same tube, making a total 5 mL LB1 added to the faecal sample. The mixture was vortexed and incubated at 60°C for 3 h, with occasional mixing for cell lysis. After centrifuging at 3,150 *g* (4,000 rpm) for 12 min, 4 mL of supernatant was transferred into a new 15 mL tube and equal volume of phenol: chloroform: isoamyl alcohol (PCI, 21:20:1) was added to the supernatant, and gently mixed. The mixture was centrifuged as above, and 3 mL of the aqueous phase was transferred into a new 15 mL tube. Next, 330 μL of Lysis Buffer 2 (LB2: CTAB 10%, NaCl 0.5 M, pH 5.5) was added to the supernatant, and incubated at 60°C for 4 h, with occasional mixing, for further lysis. Thereafter, 104 μL of protease (#P5147, Sigma Aldritch, USA) was added to the lysate to digest proteins for 1 h at 60°C. Then, equal volume (3434 μL) of PCI was added to the mixture, gently mixed, and centrifuged as above. Three mL of the aqueous phase was transferred into a new 15 mL tube and equal volume of isopropanol was added for overnight DNA precipitation at -20°C. The sample was centrifuged for 20 min at 8000 *g* (5,200 rpm), and all supernatant was decanted. The pellet was washed once with 400 μL of 70% ethanol. After being vortexed and centrifuged at 3,150 *g* for 12 min, the supernatant was decanted. The pellet was air dried at room temperature for 15 min and resuspended in 250 μL of TE buffer (10 mM Tris–HCl, 1 mM EDTA, pH 8). A step-by-step version of this protocol can be found in the protocol.io repository [43].

#### DNA extraction from skin

DNA was extracted using DNeasy Blood and Tissue kit (#69504, Qiagen, Germany). Approximately 10 mg of dugong skin tissue was ground to fine powder using liquid N_2_, and 180 μL of Buffer ATL was added into the mortar for further grinding. The mixture was transferred into a 1.5 mL tube, and the addition of another 50 μL of Buffer ATL to the mortar aided recovery of any remaining tissue. The rest of the extraction followed manufacturer’s protocol, using a 3 h incubation period, with 100 μL elution in a 1.5 mL tube. A centrifuging step was added to the protocol following the addition of Buffer AL to remove cellular debris, and 99.9% ethanol was added to the supernatant for DNA precipitation.

#### DNA quantification

Using 2 μL of DNA extract, concentration (ng/μL) and purity (A260/280 and A260/230) of all extracted DNA were measured using NanoDrop™ 1000 Spectrophotometer (Thermo Fisher Scientific, USA). DNA isolates were stored at -80°C until use.

#### Real-time PCR assays

Quantitative PCR (qPCR) was conducted for all markers in a CFX96 Touch Real-Time PCR Detection System (Bio-Rad, USA), using 96-well PCR plates, and a ‘no template control’ (NTC) was used to detect contamination. DNA was replaced with nuclease-free water in NTC, and PCR results were voided when there was presence of contamination. DNA extracted from tissue was used as positive control. Melt curve analysis was performed to determine melt temperature of the primers and to detect presence of non-specific amplifications.

#### mtDNA and ZFX markers

The control region of mtDNA and the zinc finger X-chromosomal protein encoding ZFX gene of nDNA were amplified using specific primers developed by Tol *et al*. [44] and McHale *et al*. [45], respectively (Table 2). These markers were used throughout the entire study except for SNP amplifications.

**Table 2:** Forward and reverse dugong primer sequences for mtDNA control region and ZFX marker used in this study, with their corresponding amplicon sizes and annealing temperatures.

Locus	Forward primer sequence (5’-3’)	Reverse primer sequence (5’-3’)	Amplicon size (bp)	Annealing temp (°C)	Reference
**5’ end control region**	CGCGCGCTATGTACTTCGT	GGGGTAAGTAGTGTAATGCACG	110	65	[44]
**ZFX**	AAGCATAGTAAAGAGATGCCGT	ATGTCACACTTGTGGGGGTA	230	58	[45]

PCRs were performed with a 20 μL reaction volume, each consisting of a variable amount of total DNA (Table 3), variable concentration of forward and reverse primers (Table 3), nuclease-free water, and 10 μL of PowerUp™ SYBR™ Green Master Mix (Applied Biosystems, USA).

**Table 3:** Summary of the total DNA (ng) and concentrations of forward and reverse primers (μM) added to the PCR reaction for the corresponding experiments and DNA extraction methods.

DNA extraction method	Usage	Primer	Total DNA added (ng)	Concentration of forward and reverse primers used (μM)
**QIAamp**	Compare faecal sampling techniques	mtDNA	50	0.1
**QIAamp**	Develop DNA extraction method, Compare faecal layer sampling on faeces of different quality	mtDNA	100	0.1
**QIAamp**	Develop DNA extraction method	ZFX	100	0.5
**HV-CTAB-PCI**	Develop DNA extraction method, Compare faecal quality	mtDNA	Undiluted total DNA /2	0.1
**HV-CTAB-PCI**	Develop DNA extraction method, Compare faecal quality	ZFX	Undiluted total DNA /2	0.3
**HV-CTAB-PCI**	SNP amplification	SNP	Undiluted total DNA /2	0.5

When DNA extracted with QIAamp method was used in the PCRs, the amount of total DNA added to the reactions was standardised and served as a normaliser to enable comparisons between samples. For the comparison of sampling from different layers of faeces of different EELs, a pilot experiment was conducted to determine whether 10 or 100 ng of total DNA should be used in the reactions (S1 Fig). As eroded faeces appeared to have higher PCR success for amplification of mitochondrial marker using 100 ng of total DNA, this amount was chosen, although the difference in amplification success was not statistically significant. When DNA extracted with HV-CTAB-PCI method was used, a 1:2 dilution factor provided the highest and most consistent amplification of all markers in the dilution experiments and was thus used for all subsequent PCR reactions.

Cycling conditions were 50°C for 2 min, then 95°C for 2 min, followed by 45 cycles of 95°C for 15 s, respective annealing temperature (Table 2) for 1 min, and ending with a melt profile from 65°C to 95°C, with 0.1°C increments.

**SNP markers.** The 10 SNP primers used were developed by McGowan *et al*. [29] (Table 4). PCRs were performed as described previously. Total DNA diluted 1:2 was added to each reaction, and 0.5 μM of forward and reverse primers were used (Table 3).

**Table 4:** Forward and reverse sequences of SNP primers used in this study, with their corresponding amplicon sizes and annealing temperatures.

SNP Locus	Forward primer sequence (5’-3’)	Reverse primer sequence (5’-3’)	Amplicon size (bp)	Annealing temp (°C)	Reference
**Dug12**	TCAGAAACAGAGCAACCAAAAA	CTTTGGAGTCTGAGCTCTGGA	114	60	
**Dug40**	TGATGAGCTAAACTAAAACAGAAACC	TGTTTCATGAGGTTAAGGGGA	114	60	
**Dug50**	GGGCTCCAGCTCTCACTGTA	GGTGATTATCAAACTTCTTTCCAGA	107	60	
**Dug51**	ACCATGACCAAGTGGGATTC	AAAACAAAATACAAGAACCACATGA	109	60	
**Dug53**	GATCTGTTGCGTTGCTCTTG	TCTATTTCAGGGCTGGCATG	104	60	[29]
**Dug54**	GCAGGCTGCACAATTAACTTTTA	GAGCACCCGTAAAGGGAAAC	123	60	
**Dug60**	TGCCCAGATTTCTAGCTTGG	TCTCAGTTAGTGGCAACTCCA	116	60	
**Dug62**	AAGCATCAAAGGAAGATGCAA	GGTAGCATATGATCAGGAACCG	101	60	
**Dug63**	TTCAAAGTTGACTGATAGTGAGAAA	GCTGTGCTAATTCTGGGGTC	121	60	
**Dug64**	TGTAGGAAGTCGGGAGTTGG	TTTATTCCCCACTGCGATTC	101	60	

Cycling conditions were 50°C for 2 min, 95°C for 2 min, followed by 45 cycles of 95°C for 15 s, 60°C for 30 s, 72°C for 30 s, and 72°C for 15 min. A melt profile from 65°C to 95°C, with 0.5°C increments was performed.

### qPCR intra-assay and inter-assay variability

Quantitative PCR reactions were performed in triplicate to account for intra-assay variability, and the results were averaged for final analysis. To account for inter-assay variability, threshold cycle (C_T_) was manually set to the same value using the CFX Maestro software (Bio-Rad, USA) when C_T_ values were compared, and tissue control was used as an inter-plate calibrator in each plate when relative quantity was calculated and compared.

### Primer efficiency and linearity

To assess the qPCR assay performances, standard curves for both mtDNA and ZFX primers were established through seven 10-fold dilutions of two skin DNA isolates, starting from 1000 ng of total DNA. PCR efficiency (*E*) of each primer was calculated from the slope of the dilution curve as per Ruijter *et al*. [46].

Dilution curves for mtDNA and ZFX primers were also generated using five 10-fold serial dilutions and four 10-fold serial dilutions of two fresh faecal DNA extracts (*ex*-EE-L1), respectively, starting from five times the undiluted total DNA. The *E* of both primers were determined from these dilution curves and compared to the *E* of the purer skin DNA matrix. As part of the development of HV-CTAB-PCI protocol, the dilution concentration that enabled optimal amplification (i.e., produced lowest C_T_ values) was chosen as the concentration used in PCR reactions. Similar dilutions for both primers were also trialled using the most eroded faecal type (i*n*-EE-L5) to confirm that similar dilution concentration also worked optimally in these faeces. This concentration was verified to be higher than the limit of detection (LOD) to ensure the validity of results. The LOD of both primers was determined as the lowest amount of total DNA that produced at least 95% of positive amplifications in the replicates of a faecal sample [47]. Standard curves, from which *E* were determined, were created using the range of total DNA that produced linear results, i.e., extreme concentrations that led to non-accurate estimates of *E* were not included. The coefficient of determination (R^2^), that indicates the goodness of fit, was determined for all standard curves.

### Primer specificity

Unpurified PCR products were submitted to the Australian Genome Research Facility (AGRF) at The University of Queensland, Australia, for purification and dual-direction Sanger sequencing. Sequences from mtDNA and ZFX amplifications underwent BLAST searches on GenBank, and sequence similarity was used to confirm specific amplification of respective dugong DNA. The resultant SNP sequences were compared to amplicon sequences of McGowan *et al*. [29] to confirm amplification of interested regions, and to determine the SNP allele possessed by the individual dugongs. Specificity of PCR amplicons was additionally verified through a melt curve analysis for each PCR reaction. SNP sequences were aligned and trimmed using MEGA11 software [48].

### Data processing

As C_T_ value is inverse to the amount of target DNA present within a sample, the inverse of C_T_ value was used for comparative analyses to facilitate visualisation of results. Relative quantity of target mtDNA was calculated in the CFX-Maestro software (Bio-Rad, USA), using a tissue sample as control and *E*. The formula from the software’s user guide (version 1.1) used was:

$${Relative\;Quantity}_{sample(mtDNA)}={E_{mtDNA}}^{\left(C_{T(control)}-C_{T(sample)}\right)}$$,

where *E*_*mtDNA*_ represents the *E* of mtDNA primer, and *C*_*T(control)*_ and *C*_*T(sample)*_ represent the average C_T_ value for the control and sample, respectively. The relative quantity was multiplied by 100,000 for easier interpretation of small numbers. For samples that failed to amplify, a C_T_ value of the number of PCR cycles used + 1 was assigned to enable the determination of lowest possible C_T_ value difference for comparison.

### Statistical analyses

All statistical analyses were performed using *R* (version 4.2.0, [49]). For all parametric models fitted, diagnostic plots were made to check whether the assumptions for homogeneity and normality of residual variance were met. Normality was further confirmed with the Shapiro-Wilk’s test. Data were log-transformed when residuals were not normal. When significant difference was found in the initial model, a post-hoc pairwise Tukey test was performed using *lsmeans* package for further pairwise analyses. False discovery rate adjusted p-values were obtained when non-parametric tests were used. Graphs were created using *ggplot2* [50], *dplyr*, *hrbrthemes* [51], *Rmisc* [52], *ggpmisc* [53], *reshape*, *ggsignif* [54], and *ggpubr* [55] packages.

A linear mixed effects model (LMEM) from *nlme* package [56] was used to compare total DNA concentration and inverse C_T_ between different faecal sampling techniques, where random effects of individuals within PCR plates were accounted for. It was also used to compare log total DNA concentration recovered between different DNA extraction methods, accounting for the random effects of individuals between PCR plates. For all primers, PCR success (binary outcome: successful—if one or more amplified versus unsuccessful—none amplified) between DNA extraction methods was compared using Pearson’s Chi-square test (χ^2^), with a Monte Carlo simulation utilising 10,000 replicates, followed by post-hoc pairwise Chi-square tests. The number of amplifications within a triplicate (i.e., triplicate success: 0, 1, 2, or 3) for all primers, was compared between DNA extraction methods using Kruskal-Wallis test and post-hoc pairwise Wilcoxon rank sum (*W*) test.

To determine the effects of environmental exposure and layers of faeces sampled on the amplification of dugong DNA, a full interactive generalised linear model (GLM) with all variables (sampling layer, faecal EELs, year of collection, individuals) was fitted to compare the total DNA concentration and relative quantity of dugong mtDNA. The Akaike Information Criterion (AIC) or stepAIC function from *MASS* package [57] was used to determine significant variables through a dual-direction stepwise model selection. A final additive LMEM was fitted to compare total DNA concentration and log relative quantity of dugong mtDNA between faecal sampling layers and faecal EELs, accounting for random effects of different individuals. The relationship between PCR success and log total DNA was tested using a quadratic GLM with binomial responses.

A likelihood ratio Chi-square test (LR-Chi-square test) was used to compare between interactive (sampling layer*faecal EELs) and additive (sampling layer + faecal EELs) GLMs, and a final additive GLM with biased reduction was fitted, using *brglm2* package [58], to compare PCR success between the two explanatory variables. A LR-Chi-square test was also used to compare between four multinomial log-linear models (from *nnet* package; [57]) fitted with interactive, additive, and single explanatory effects on the triplicate success of samples. A post-hoc pairwise *W* test was then used to compare triplicate success between sampling layer and faecal EELs.

When HV-CTAB-PCI method was used, total DNA concentration was compared between faecal EELs using a one-way analysis of variance (ANOVA), whilst PCR and triplicate successes were compared between different faecal EELs using the χ^2^ and Kruskal-Wallis tests as described earlier. Similarly, the two non-parametric tests were also used to compare the PCR and triplicate successes between different primers and faecal types in SNP amplifications.

### Ethics statement

Dugong samples were obtained under The University of Queensland Animal Ethics Permit SBS/181/18, Scientific Purposes Permit WISP14654414, Moreton Bay Marine Parks Permit MPP18-001119, and Great Barrier Reef Marine Park Permit G14/36987.1.

## Results

### PCR efficiencies and LOD

**Fig 4:**
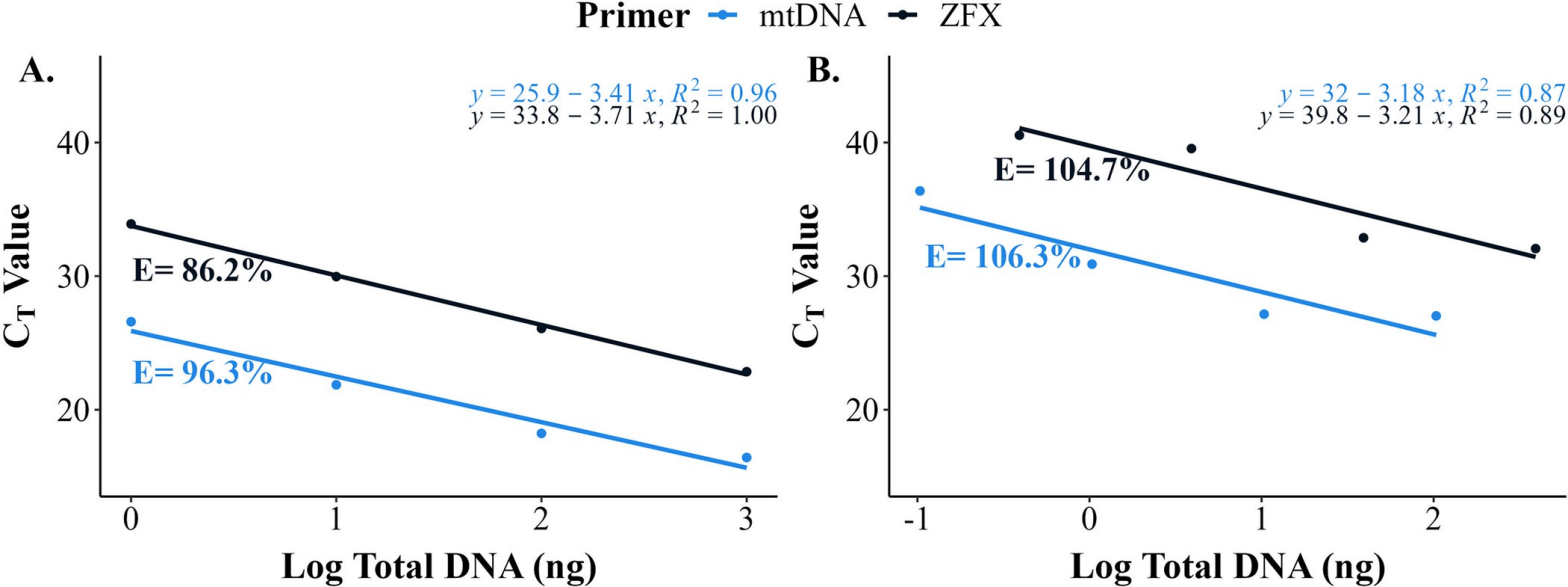
PCR dilution curves for each primer set. (A) The dilution curve of tissue DNA isolates extracted using the Qiagen DNeasy method. (B) The dilution curve of faecal DNA isolates extracted using the HV-CTAB-PCI method. An efficiency (*E*) > 100% indicated slight inhibition or low yield of DNA for both primers, using faecal DNA isolates. The initial total DNA amount (ng) used for PCR reactions was determined from these dilutions.

PCR efficiency for amplification of tissue DNA was 96.3% and 86.2% for mtDNA and ZFX primers, respectively (Fig 4A). The *E* for amplification of faecal DNA, both greater than 100%, indicated slight inhibition (mtDNA = 106.3%, ZFX = 104.7%; Fig 4B) [59], which was addressed through dilutions. The quantities of total DNA used for PCR reactions were higher than LOD for both primers (LOD for mtDNA: 1 ng, ZFX: 4 ng). Standard curves of tissue DNA had R^2^ > 0.95 which validated the primers and assays used [60]. The R^2^ values for dilution curves of faecal DNA (a mixture of target and exogenous DNA) were greater than 0.85; these were considered adequate for their purpose in this study.

### DNA extraction method comparison

**Fig 5:**
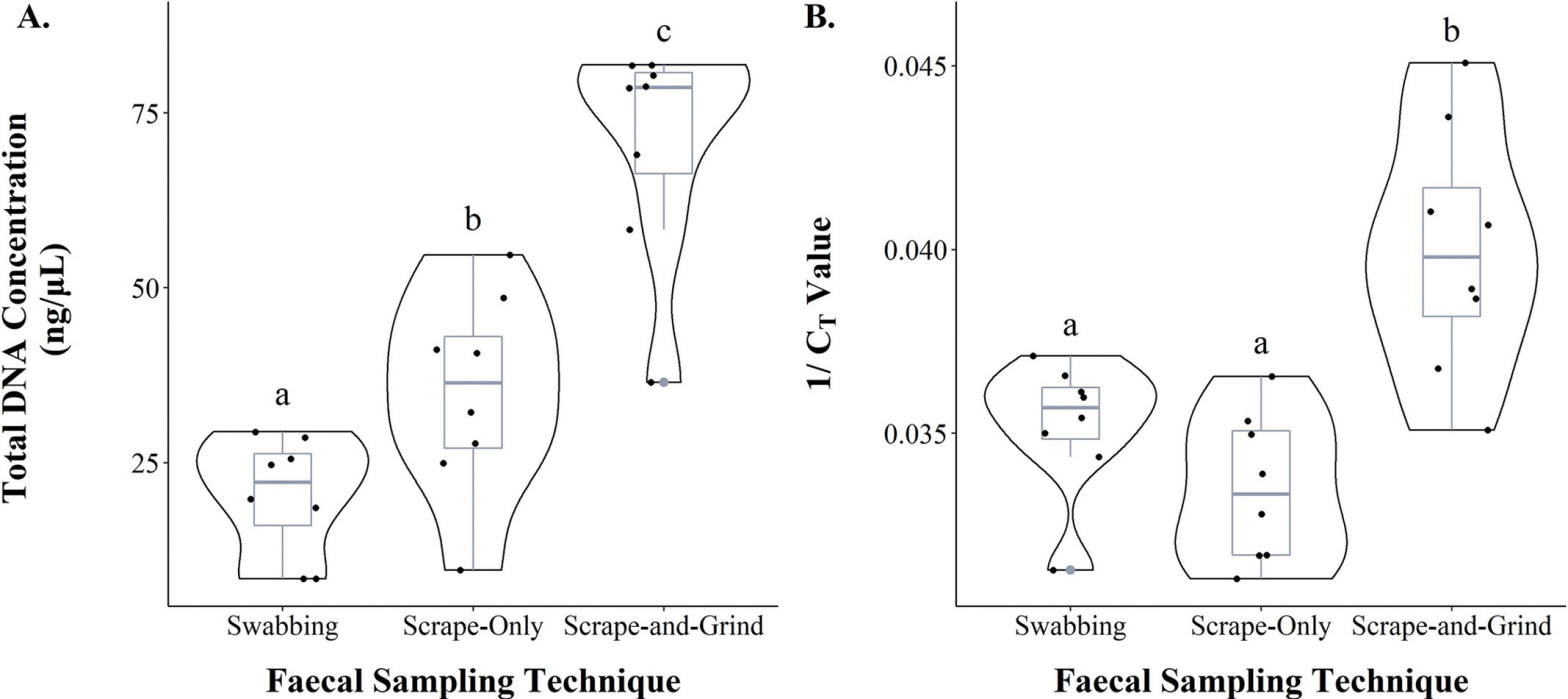
Comparison between faecal sampling and processing techniques to determine the best sampling protocol for DNA extraction. Violin plot shows the distribution of the data, whilst the box plot shows the first (Q1), second (Q2) and third (Q3) quartiles, minimum, maximum values, and outliers. All DNA was extracted using the QIAamp protocol. (A) Comparison of total DNA concentration extracted using each sampling and processing technique. (B) Comparison of the inverse C_T_ values from mtDNA amplification using faecal DNA extracted with each sampling and processing technique. Similar letters denote no statistical significance, while different letters denote statistical significance with an alpha value of 0.05.

The scrape-and-grind technique yielded significantly higher concentrations of total DNA (70.7 ± 5.3 ng/μL; mean ± standard error) compared to the scrape-only (34.9 ± 4.8 ng/μL, Tukey-HSD: T_14_ = 8.361, p < 0.001) and swabbing (20.4 ± 2.8 ng/μL, Tukey-HSD: T_14_ = 11.762, p < 0.001) techniques (Fig 5A). Total DNA concentrations, yielded using the scrape-only technique, were significantly higher than that of swabbing (Tukey-HSD: T_14_ = 3.401, p = 0.011). The scrape-and-grind technique also had higher inverse C_T_ values (0.040 ± 0.001) compared to scrape-only (0.033 ± 0.001, Tukey-HSD: T_14_ = 6.027, p < 0.001) and swabbing (0.035 ± 0.001, Tukey-HSD: T_14_ = 4.413, p = 0.002) techniques (Fig 5B). However, the inverse C_T_ values were not significantly different between scrape only and swabbing techniques (Tukey-HSD: T_14_ = 1.613, p = 0.273).

**Fig 6:**
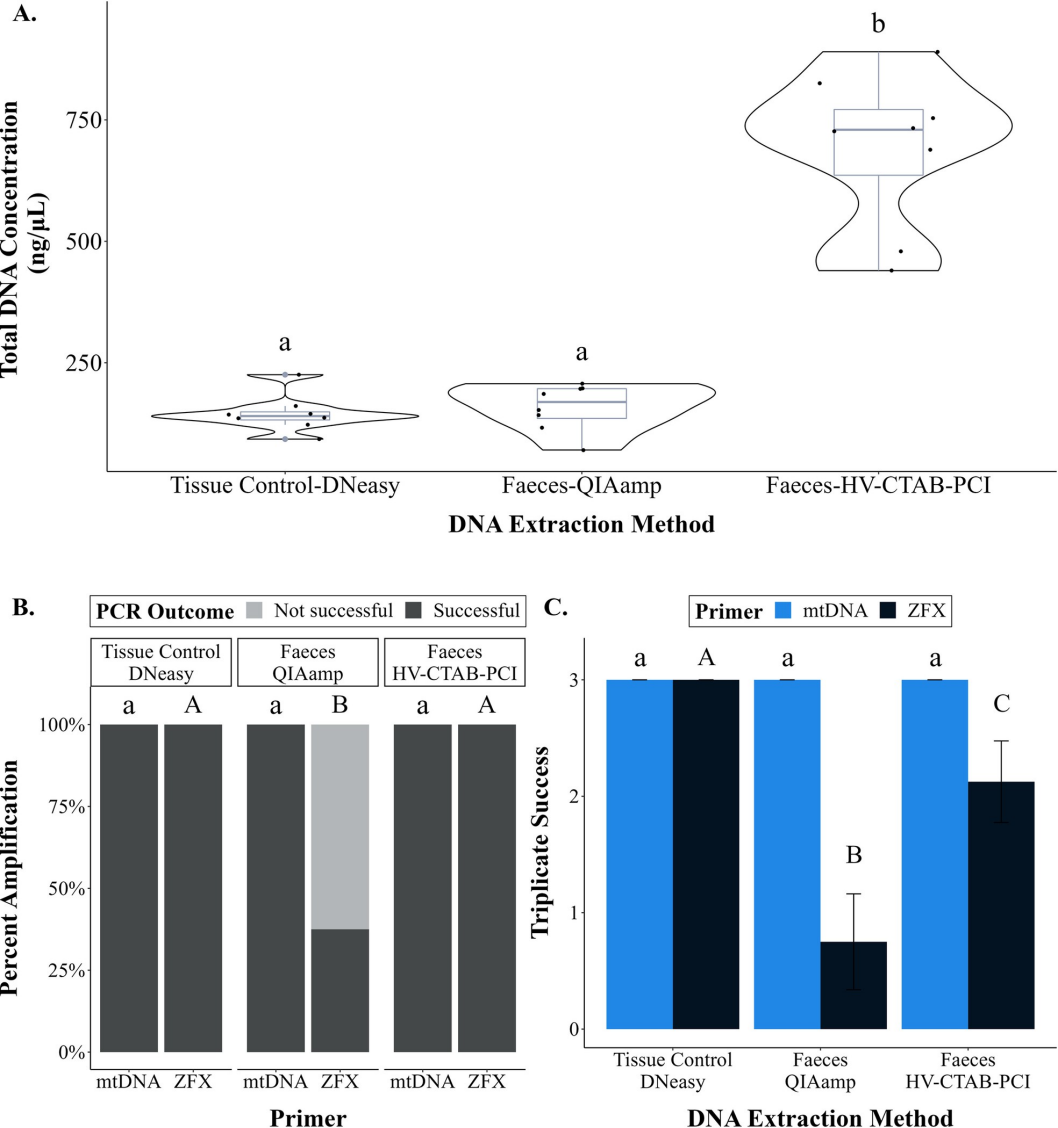
Comparisons between two DNA extraction methods and a tissue control. (A) A violin plot with box plot showing the total DNA concentration of each DNA extraction method. (B) A stacked bar graph showing the percent amplification that was successful (PCR success) and unsuccessful between all DNA extraction methods for each primer. (C) A bar graph showing the mean ± standard error of the number of replicates that amplified in triplicate for each DNA extraction method. Similar letters (with same letter case) denote no statistical significance, while different letters denote statistical significance (α = 0.05). Different statistical comparisons made in this study were indicated by different letter case.

Total DNA concentration extracted from faeces using HV-CTAB-PCI method (691.9 ± 51.9 ng/μL) was significantly higher than that extracted using QIAamp method (158.5 ± 15.8 ng/μL, Tukey-HSD: T_14_ = 11.701, p < 0.001, Fig 6A). The former method extracted more total DNA than control (145.4 ± 12.6 ng/μL, Tukey-HSD: T_14_ = 12.196, p < 0.001), but total DNA extracted using QIAamp method was not significantly different from control (Tukey-HSD: T_14_ = 0.496, p = 0.875). On average, the purity of faecal DNA extract was higher using QIAamp rather than HV-CTAB-PCI method (Table 5).

**Table 5:** Purity (A260/280 and A260/230) values of DNA extracted for each sample type using the Qiagen kits and a newly developed HV-CTAB-PCI method.

DNA Extraction Method	Sample Type	A260/280 (mean ± std error)	A260/230 (mean ± std error)
**Qiagen DNeasy**	Tissue	2.12 ± 0.01	1.82 ± 0.04
**QIAamp**	Faeces	1.95 ± 0.01	1.27 ± 0.03
**HV-CTAB-PCI**	Tissue	2.06 ± 0.01	2.14 ± 0.01
**HV-CTAB-PCI**	Faeces	1.67 ± 0.01	1.06 ± 0.03

PCR success and triplicate success for mtDNA amplification were similar between all DNA extraction methods, as all replicates of all samples amplified successfully (Fig 6B and 6C). For ZFX amplification, PCR success of HV-CTAB-PCI method was the same as control, and PCR success of those two were significantly higher than that of the QIAamp method (χ^2^ = 7.273, p = 0.026). All samples amplified in control and HV-CTAB-PCI method, but only 37.5% of faecal samples amplified in the QIAamp method (Fig 6B). The control had significantly higher triplicate success for ZFX amplification (Mean, $\bar{x}$ amplification = 3/3) compared to HV-CTAB-PCI ($\bar{x}$ amplification = 2.1/3, *W* test: Z = 1.86, p = 0.032) and QIAamp ($\bar{x}$ amplification = 0.8/3, *W* test: Z = 2.69, p = 0.004) methods (Fig 6C). However, triplicate success of HV-CTAB-PCI method was higher than that of QIAamp method (*W*-test: Z = 1.86, p = 0.032).

### Effects of EELs and faecal layers sampled

Effects of sampling from different faecal layers did not depend on faecal EELs as all interactive models had higher AIC values compared to additive models (Table 6). Same results were obtained for LR-Chi-Square test (Deviance = 0.022–0.832, p > 0.842).

**Table 6:** The AIC values obtained for interactive and additive models fitted in *R* for different variables compared using a dual-direction stepwise model selection.

Response variable	Type of model	AIC values
**Total DNA concentration**	Interactive	349.98
	Additive	346.74
**Log relative amount of mtDNA**	Interactive	930.97
	Additive	929.56

### Impacts of faecal layer sampled

**Fig 7:**
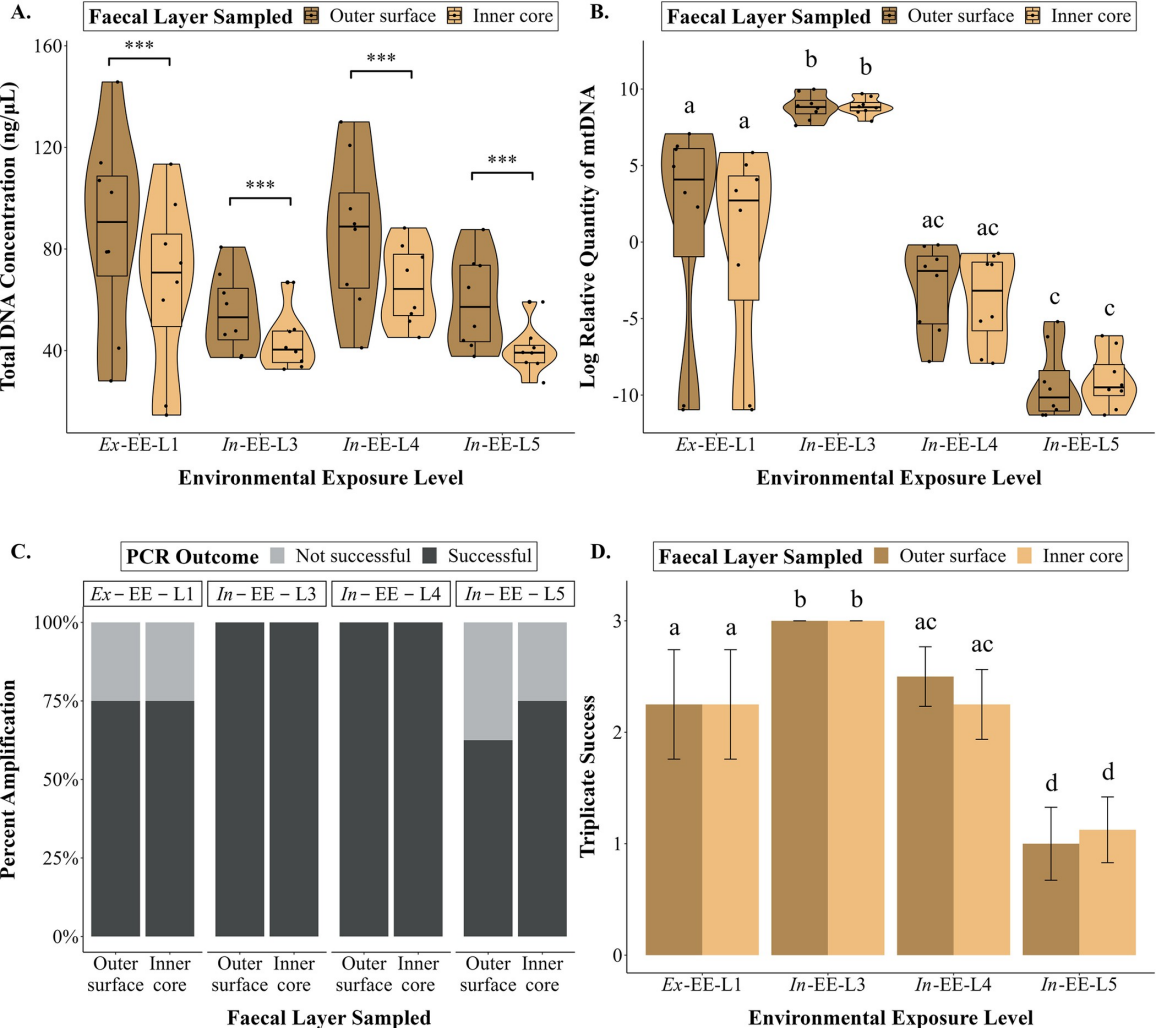
Comparison of the variables measured between outer surface and inner core of faeces across different environmental exposure levels (EELs) representing varying quality. All DNA was extracted using the QIAamp method. (A) Total DNA concentration extracted from different layers of various faeces. No statistical significance was present between the faecal types, but the outer surface yielded a higher concentration of total DNA compared to the inner core for all faeces. (B) Log relative quantity of dugong mtDNA amplified from different layers of various faeces. (C) The percent mtDNA amplification that was successful and unsuccessful between different layers of various faeces. PCR success was statistically similar between all faecal types. (D) Triplicate success between different layers of various faeces. Similar letters denote non-significance, while different letters denote statistical significance (α = 0.05).

For faeces of all EELs, the total DNA concentration extracted from the outer surface of faeces was significantly higher than that extracted from their inner core (Tukey-HSD: T = 5.601, p < 0.001 for all faeces; Fig 7A). The outer surface of *ex*-EE-L1 faeces had at least 2.87× more mtDNA than their inner core, while the outer surface of *in*-EE-L3, L4, and L5 faeces had at least 0.14×, 0.65×, and 0.95× more mtDNA than their inner core, respectively (Fig 7B). On average, the outer surface of all stools had at least 0.16× more dugong mtDNA than the inner core of faeces. However, none of the differences were significant (Tukey-HSD: T = 1.222, p = 0.919 for all faeces). PCR success (Biased-Reduction-GLM: Z = 0.373, p = 0.709; Fig 7C) and triplicate success (Likelihood-ratio = 0.832, p = 0.842; Fig 7D) of mtDNA amplification were the same regardless of whether outer surface or inner core of faeces were sampled. As the layer of faeces sampled had no significant effect on the amplification success, only the outer surface of faeces was used for further comparisons.

### Impacts of environmental exposure

When the QIAamp method was used, the total DNA concentration extracted from *ex*-EE-L1 (87.0 ± 12.8 ng/μL), *in*-EE-L3 (55.2 ± 5.1 ng/μL), *in*-EE-L4 (86.5 ± 10.0 ng/μL), and *in*-EE-L5 (59.2 ± 6.06 ng/μL) faeces were not significantly different from each other regardless of the faecal layer sampled (Tukey-HSD: -2.877 < T_28_ < 0.970, p > 0.123; Fig 7A). The amount of dugong mtDNA extracted from *ex*-EE-L1 faeces was at least 46× less than that extracted from *in*-EE-L3 faeces (Tukey-HSD: T_30_ = -4.548, p < 0.001), but was 247,253× higher than that isolated from *in*-EE-L5 faeces (Tukey-HSD: T_30_ = 5.105, p < 0.001) (Fig 7B). Amount of dugong mtDNA was not significantly different between *ex*-EE-L1 and *in*-EE-L4 faeces (Tukey-HSD: T_29_ = 1.910, p = 0.246). However, amount of dugong mtDNA in *in*-EE-L3 faeces was at least 38,322× and 11,697,397× higher than that of the *in*-EE-L4 (Tukey-HSD: T_29_ = 6.604, p = < 0.001) and *in*-EE-L5 (Tukey-HSD: T_30_ = 14.405, p = < 0.001) faeces, respectively. The amount of dugong mtDNA was the same in *in*-EE-L4 and L5 faeces (Tukey-HSD: T_29_ = 3.049, p = 0.081).

PCR success for mtDNA amplification was similar between faeces of all EELs (Tukey-HSD: -1.583 < Z < 1.775, p > 0.285; Fig 7C). Despite insignificant results, all *in*-EE-L3 and L4 faeces amplified, but only 75% of *ex*-EE-L1 and 68.8% of *in*-EE-L5 faeces amplified successfully. However, *in*-EE-L3 faeces had higher triplicate success ($\bar{x}$ amplification = 3/3) compared to *ex*-EE-L1 ($\bar{x}$ amplification = 2.3/3, *W*-test: Z = 1.680, p = 0.046), *in*-EE-L4 ($\bar{x}$ amplification = 2.4/3, *W*-test: Z = 2.530, p = 0.006) and *in*-EE-L5 faeces ($\bar{x}$ amplification = 1.1/3, *W*-test: Z = 4.68, p < 0.001) (Fig 7D). Triplicate success of *ex*-EE-L1 faeces was not significantly different compared to that of *in*-EE-L4 faeces (*W*-test: Z = 0.444, p = 0.672) but was significantly higher than that of *in*-EE-L5 faeces (*W*-test: Z = 2.530, p = 0.006). The *in*-EE-L4 faeces also had a higher triplicate success than *in*-EE-L5 faeces (*W*-test: Z = 3.011, p = 0.001).

**Fig 8:**
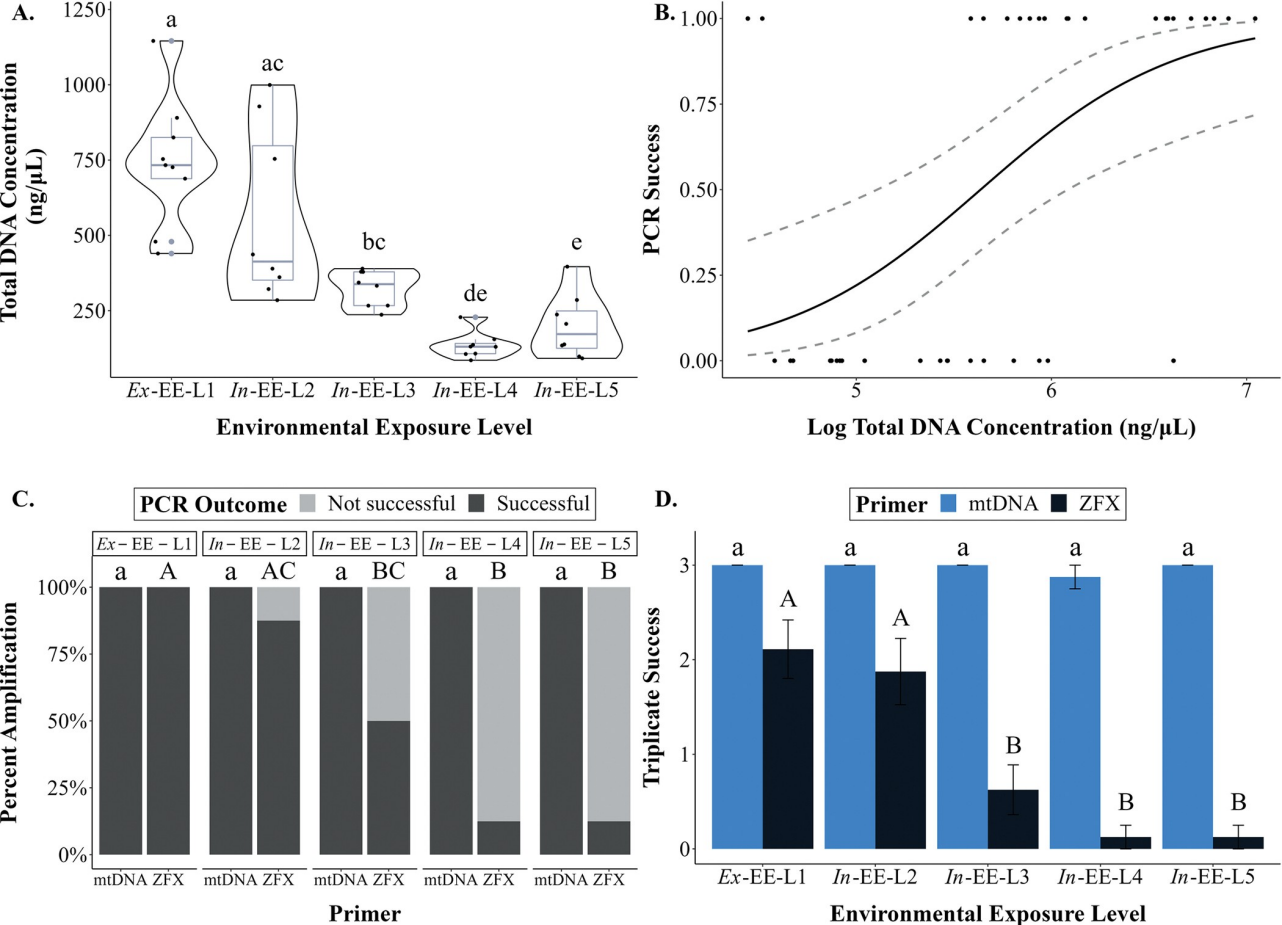
Comparison of variables measured in faeces with different environmental exposure levels (EELs) representing varying quality. All DNA was extracted using the HV-CTAB-PCI method. (A) Total DNA concentration extracted from each faecal type. (B) Graph shows a positive correlation between PCR success and total DNA concentration. Lines represent mean ± standard error. (C) Percent amplification that was successful and unsuccessful for mtDNA and ZFX primers between different faecal types. (D) Triplicate success between the faecal types for both primers. Similar letters (with same letter case) denote no statistical significance, while different letters denote statistical significance (α = 0.05). Different statistical comparisons made in this study were indicated by different letter case.

When the HV-CTAB-PCI method was used, the total DNA concentration extracted from *ex*-EE-L1 faeces (742.3 ± 70.3 ng/μL; mean ± standard error) was not significantly different to that extracted from *in*-EE-L2 faeces (559.4 ± 95.4 ng/μL, ANOVA: T_36_ = 1.934, p = 0.318), but was significantly higher than that extracted from *in*-EE-L3 (324.1 ± 19.8 ng/μL, ANOVA: T_36_ = 4.372, p = 0.001), *in*-EE-L4 (134.8 ± 14.4 ng/μL, ANOVA: T_36_ = 9.253, p < 0.001) and *in*-EE-L5 (198.3 ± 34.8 ng/μL, ANOVA: T_36_ = 7.597, p < 0.001) faeces (Fig 8A). Total DNA concentration of *in*-EE-L2 faeces was not significantly different compared to that of *in*-EE-L3 faeces (ANOVA: T_36_ = 2.369, p = 0.147), but was significantly higher that than of *in*-EE-L4 (ANOVA: T_36_ = 7.113, p < 0.001) and *in*-EE-L5 (ANOVA: T_36_ = 5.503, p < 0.001) faeces. The *in*-EE-L3 faeces also had higher concentration of total DNA compared to *in*-EE-L4 (ANOVA: T_36_ = 4.744, p < 0.001) and *in*-EE-L5 (ANOVA: T_36_ = 3.134, p = 0.027) faeces, but total DNA concentration between *in*-EE-L4 and L5 faeces was not significantly different (ANOVA: T_36_ = 1.610, p = 0.501).

PCR success and triplicate success (Kruskal-Wallis: χ^2^ = 4.125, p = 0.389) for mtDNA amplification were the same across faeces of all EELs, as all samples amplified successfully (Fig 8C and 8D). Mean triplicate success for faeces of all EELs was 3, except for *in*-EE-L4 faeces which was 2.9. For ZFX amplification, faeces with higher total DNA concentration tended to have higher PCR success (GLM: Z = 2.558, p = 0.011; Fig 8B). PCR success of *ex*-EE-L1 faeces was similar to that of *in*-EE-L2 faeces (χ^2^ = 1.195, p = 0.471), but was significantly higher than that of *in*-EE-L3 (χ^2^ = 5.885, P = 0.032), *in*-EE-L4 (χ^2^ = 13.39, p< 0.001), and *in*-EE-L5 (χ^2^ = 11.46, p = 0.003) faeces (Fig 8C). All *ex*-EE-L1 faeces amplified, but only 87.5% and 50.0% of *in*-EE-L2 and L3 faeces amplified, respectively. Only 12.5% of both *in*-EE-L4 and L5 faeces amplified. PCR success of *in*-EE-L2 faeces was not significantly different compared to that of *in*-EE-L3 faeces (χ^2^ = 2.618, p = 0.283), but was higher than that of *in*-EE-L4 (χ^2^ = 9.00, P = 0.012) and *in*-EE-L5 faeces (χ^2^ = 7.244, p = 0.015). PCR success of *in*-EE-L3, L4, and L5 faeces was not significantly different from one another (0.275 < χ^2^ < 2.618, p> 0.284). Triplicate success of *ex*-EE-L1 faeces ($\bar{x}$ amplification = 2.1/3) was similar to that of *in*-EE-L2 faeces ($\bar{x}$ amplification = 1.9/3, *W-t*est: Z = 0.711, p = 0.761), but higher than that of *in*-EE-L3 ($\bar{x}$ amplification = 0.6/3, *W-*test: Z = 2.197, p = 0.014), *in*-EE-L4, and L5 (both $\bar{x}$ amplification = 0.1/3, both *W-*tests: Z = 2.759, p = 0.003) faeces (Fig 8D). The *in*-EE-L2 faeces also had a higher triplicate success compared to *in*-EE-L3 (*W-*test: Z = 1.794, P = 0.036), *in*-EE-L4, and L5 (both *W-*tests: Z = 2.473, p = 0.007) faeces, but triplicate success of *in*-EE-L3, L4 and L5 faeces were not significantly different from each other (*W-*tests: Z < 1.029, p> 0.152).

### SNP amplifications in *in-ōceanum* faeces

**Fig 9:**
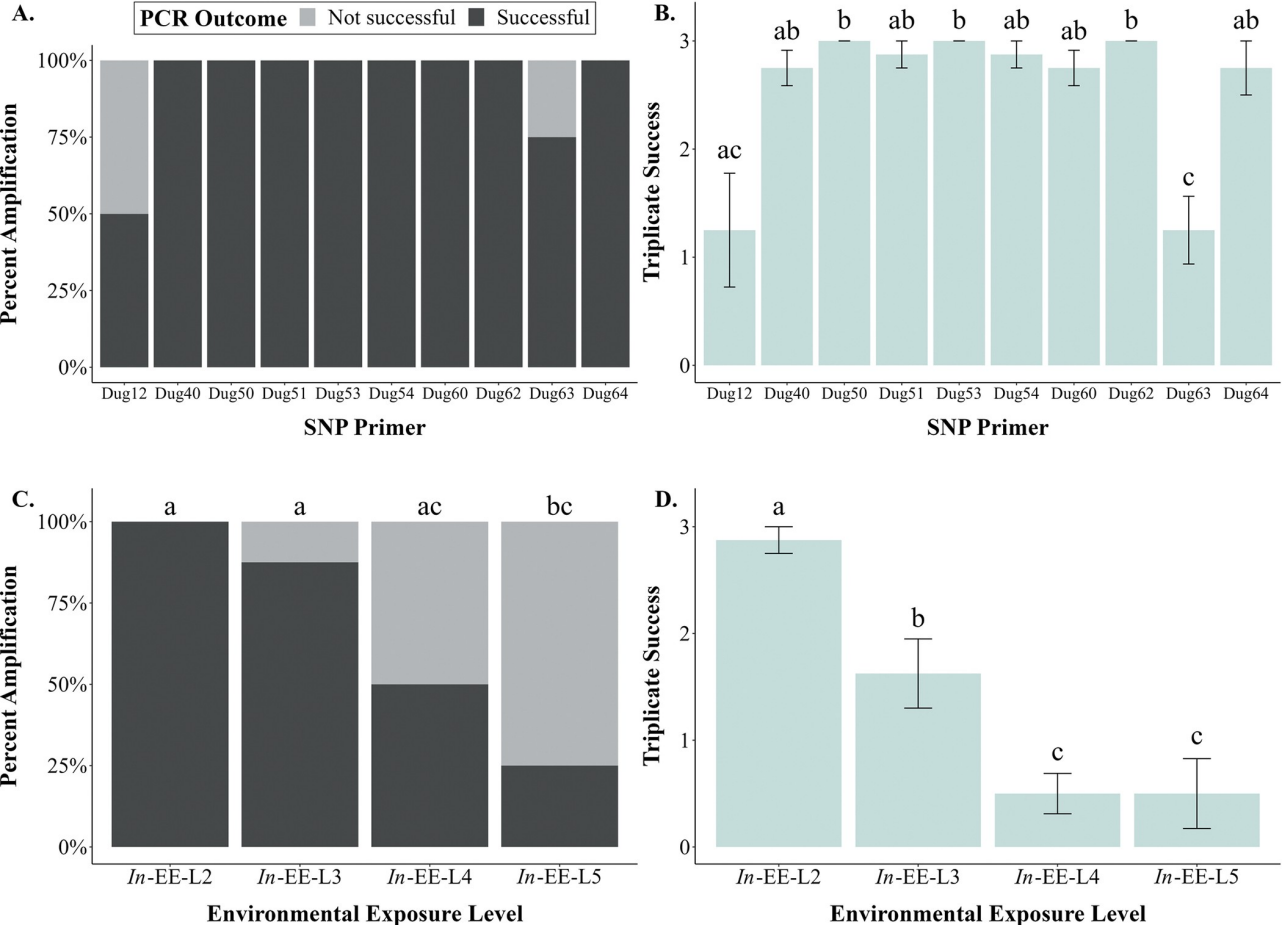
PCR success and triplicate success between SNP primers and faeces with different environmental exposure levels (EELs), representing its quality. All DNA was extracted using the HV-CTAB-PCI method. (A) PCR success of different SNP primers amplified in all *in*-EE-L2 faeces. No statistical significance exists between primers used. (B) Triplicate success of different SNP primers amplified in all *in*-EE-L2 faeces. (C) PCR success of primer Dug54 between faeces of different EELs. (D) Triplicate success of primer Dug54 between faeces of different EELs. Similar letters denote no statistical significance, while different letters denote statistical significance (α = 0.05).

All *in*-EE-L2 faecal samples amplified for all dugong SNP primers, except primers Dug12 and Dug63, for which 50% and 75% of all samples amplified, respectively (Fig 9A). The SNP regions were successfully located and aligned, showing the form of allele possessed (S2 Fig). However, PCR success was not significantly different between amplification of different primer sets (χ^2^ < 5.33, p> 0.079; Fig 9A). Triplicate success of primer Dug12 ($\bar{x}$ amplification = 1.3/3) was significantly lower than that of primers Dug50, Dug53, and Dug62 (All $\bar{x}$ amplifications = 3/3, *W*-tests: Z = 1.694, p = 0.045) (Fig 9B). Triplicate success of primer Dug63 ($\bar{x}$ amplification = 1.3/3) was equal to that of Dug12, but significantly lower than that of all other primers (All $\bar{x}$ amplifications > 2.8/3, *W*-tests: Z > 2.115, p < 0.017).

For amplification of SNP primer Dug54 in all *in-ōceanum* faeces, the PCR success of *in*-EE-L2 faeces was significantly higher than that of *in*-EE-L5 faeces (χ^2^ = 9.600, p = 0.008), but similar to that of *in*-EE-L3 and L4 faeces (χ^2^< 5.333, p > 0.077) (Fig 9C). All *in*-EE-L2 faeces amplified, and 87.5%, 50.0%, and 25.0% of *in*-EE-L3, L4, and L5 faeces amplified, respectively. The *in*-EE-L3 faeces also had a higher PCR success compared to *in*-EE-L5 faeces (χ^2^ = 6.349, p = 0.042), but it had a similar PCR success as *in*-EE-L4 faeces (χ^2^ = 2.618, p = 0.283). The PCR success of *in*-EE-L4 and L5 faeces was not significantly different (χ^2^ = 1.067, p = 0.603). The triplicate success of *in*-EE-L2 faeces ($\bar{x}$ amplification = 2.9/3) was significantly higher than that of the *in*-EE-L3 ($\bar{x}$ amplification = 1.6/3, *W-*test: Z = 2.382, p = 0.009), *in*-EE-L4, and L5 faeces (both $\bar{x}$ amplification = 0.5/3, both *W-*tests: Z = 2.929, p = 0.002) (Fig 9D). The *in*-EE-L3 faeces also had higher triplicate success compared to *in*-EE-L4 (*W-*test: Z = 1.937, p = 0.026) and *in*-EE-L5 faeces (*W-*test: Z = 1.706, p = 0.044). However, triplicate success of *in*-EE-L4 and L5 faeces was the same (*W-*test: Z = 0.438, p = 0.669).

## Discussion

This study has demonstrated successful amplifications of both mitochondrial and nuclear markers from dugong faeces using a novel, streamlined and cost-effective ‘High volume-CTAB-PCI’ (HV-CTAB-PCI) DNA extraction method. This protocol was developed for herbivorous marine mammals, as a practical and simplified alternative for DNA isolation using large volumes of faecal sample. The results of this study indicated that fresh faeces should be collected whenever possible, and that the entire faecal mass may be utilised for DNA extraction rather than just the outer layers. Amplification success of mtDNA was found to be similar regardless of whether faecal material was sampled from the outer surface or inner core of a stool. However, the period to which faeces were exposed to the environment had significant impacts, particularly on amplification success of nDNA markers. Although the amplification success of mtDNA was adequate with the QIAamp method, the use of HV-CTAB-PCI resulted in improvements, especially for eroded faeces (*in*-EE-L5). The nuclear marker (ZFX) failed to amplify when DNA was extracted using the QIAamp method, but the use of HV-CTAB-PCI which enabled its amplification showed higher amplifications in fresher faeces than those that were eroded. Therefore, the hypothesis suggesting greater amounts of target DNA from the outer layers of dugong stools was rejected; the hypothesis suggesting higher efficacy of amplification in fresher faeces was supported by these results. Through the successful amplification of SNP markers, this study also showed a proof-of-concept that nuclear markers amplified from dugong faeces could potentially be used for population genetic studies.

### Development of the HV-CTAB-PCI method

When DNA was extracted using the QIAamp protocol, the nuclear ZFX marker did not amplify consistently even for the freshest (*ex*-EE-L1) faeces, despite the highly successful amplification of mtDNA. On average, only 0.8 of the triplicates amplified in 37.5% of samples, and the amplification was not reproducible. This aligns with Takoukam Kamla’s [34] results showing that nuclear markers were not successfully amplified from manatee scats, using the QIAamp protocol without additional purification and pre-amplification steps. The lack of success with nDNA marker amplification in this study may be attributable to two main issues faced when retrieving target DNA from faeces.

The first issue pertains to the overall dearth of target DNA in faeces. To overcome this, Takoukam Kamla [34] performed pre-amplification to enhance the amount of manatee DNA, leading to improvements in subsequent PCRs. This method was trialled in pilot experiments of this study, but ZFX amplification was still unsuccessful, which may suggest that there is scant DNA in dugong faeces. If the amount of dugong-specific DNA originally extracted was of a negligible proportion compared to the robust quantities of exogenous DNA, then such a dilute presence of dugong DNA would render an infinitesimal probability that dugong DNA is pipetted into each well of the PCR plates [61]. Without the presence of dugong DNA in initial PCR reaction, pre-amplification becomes irrelevant. Another strategy to enhance the yield of target DNA is to increase the amount of faeces used in DNA extraction. This study utilised only 220 mg of dugong faeces for DNA extraction with the QIAamp protocol as this is the maximum amount recommended by the manufacturer. Despite the retention of a higher volume of supernatant that contains DNA and the increase in subsequent volumes of extraction buffers used, this method did not isolate sufficient dugong DNA for consistent ZFX amplification. The QIAamp Fast DNA Stool Mini Kit has another protocol for DNA extraction using larger volumes of faeces. Using this method, 10 mL of InhibitEX buffer would be added to 1 g of faeces, and 2 mL of the lysate would be used for further extraction following a similar protocol used in this study. Since target DNA could also equally be present in the other 8 mL of the lysate, five rounds of extractions would need to be performed to maximise the amount of target DNA extracted from 1 g of faeces. In cases where a limited amount of target DNA is present within faeces, as in dugongs, large volumes of faeces must be extracted, and the use of this protocol becomes laborious and less affordable. Additionally, the sheer number of washing steps required due to the small size of QIAamp spin columns included in the kit may also lead to higher loss of target DNA [62]. Takoukam Kamla [34] was able to increase the amount of faeces used (300 – 1260mg) without making the corresponding increases in volumes of any reagents, but that might have contributed to the lower purity of his DNA extracts, since there may have been insufficient reagents to efficiently clean and protect the extracted DNA.

The second issue faced with faecal DNA extraction involves potential presence of PCR inhibitors. Takoukam Kamla [34] addressed this problem by performing an extra purification step post-DNA isolation with a PCR inhibitor removal kit. The consistent amplification of dugong mtDNA and similar results from dilution experiments indicated low levels of PCR inhibition. Consequently, an additional purification step was not utilised in this study, in an attempt to minimise DNA loss [63]. Furthermore, this study showed a positive correlation between concentration of total DNA extracted and PCR success, which again hinted at a problem of scarcity of DNA in dugong faeces rather than issues of PCR inhibitors. As dugongs feed on a different and more restricted diet [37] compared to manatees [35], it is possible that there are fewer and less diverse plant secondary metabolites (e.g., tannins) to inhibit PCR in dugong faeces. Additionally, unlike the open ocean where dugongs are found, the estuarine and freshwater water bodies that manatees often inhabit can contain high concentrations of tannins due to the decomposition of plant material from the surrounding forests and mangroves. Furthermore, dugongs generally have low fibre diets compared to manatees [37] and the surface of dugong faeces is consequently smoother and less fibrous. This may affect the relative quantity of epithelial cells found within dugong faeces, as the rougher scats of manatees may stimulate more mucus secretion that entraps exfoliated cells [14].

To enable the amplification of nuclear markers from dugong faeces, a DNA extraction method that allows isolation from large volumes of faeces was required. Although such extraction was technically possible using the QIAamp method following a major up-scaling, the extensive steps required to maximise DNA retrieval and the potential need for additional purification post-DNA isolation rendered it sub-optimal. A streamlined and cost-effective approach would be more desirable and/or practical, especially since faecal samples from a large number of individuals would need to be processed for population genetic studies. This is an important consideration in localities where cost may be a prohibitive factor for such studies. Up-scaling using the QIAamp method was thus not pursued further, and a new HV-CTAB-PCI method that enabled a simple and straightforward extraction using large volumes of dugong faeces was developed. The HV-CTAB-PCI protocol was modified from Vallet *et al*’s [19] 2CTAB/PCI protocol, and the washing/ purification steps used in that method can be easily modified, without requiring extra kits, to meet the needs of this current study. Although this novel method is highly advantageous on many levels, it is important to note that phenol and chloroform are hazardous chemicals, and thus appropriate personal protective equipment and lab safety protocol (e.g., working in fume hoods) must be implemented.

The novel HV-CTAB-PCI method is markedly different to the 2CTAB/PCI approach. The minimum effective sample-to-reagent ratio used in this method (1:5) was first determined through a 1 mL-by-1 mL addition of Lysis Buffer 1 to 1 g of faecal sample. This differs from the ratios used in the 2CTAB/PCI approach [19], which would require large volumes of reagents, when scaled up, to extract DNA from 1 g of faeces. Instead of an overnight incubation, cell lysis duration was reduced to 3 h to shorten the DNA extraction procedure. To separate DNA from proteins and other impurities, an effective equal PCI-to-supernatant ratio (1:1) was utilised [64]. Proteins were digested using protease instead of the widely used Proteinase K in the HV-CTAB-PCI method as protease was found to be effective at removing proteins, and costs less than the latter enzyme. As the presence of RNA did not present problems in PCR amplifications, RNAse was not used in this newly developed protocol. To minimise DNA loss, the DNA pellet retrieved at the end of extraction was washed once only [63]. Despite a reduced washing frequency, PCR was not significantly inhibited and the amplification successes of mtDNA and ZFX were higher using this new protocol, that enabled simple large volume extractions, compared to the QIAamp method.

### Sampling from different layers of faeces of varying quality

Amplification success and relative quantity of mtDNA did not differ between DNA extracted from the outer surface and inner core of dugong stools. This result was surprising as faecal DNA extractions in most molecular scatology studies have sampled the outer surface of a scat only, e.g., [9, 15]. This current study concurs with Stenglein *et al*. [65] which found no difference in PCR success and error rates of DNA extracted from outer and inner layers of brown bear scats. Although lower allelic dropout and higher genotyping success rates were achieved when DNA from the outer surface of stools was used [65], it would be improper to conclude that most of the target animal cells are located on the outer surface as those measures only reflect the quality of DNA extracted. As the total DNA yield tended to be higher when outer surface was sampled, it is possible that the small sample size (total n = 32) in this study limited the detection of significance between outer and inner layers of faecal stools. However, it is likely that most of the DNA extracted was from non-target organisms (e.g., bacteria, plants) since the relative amount of dugong-specific mtDNA was the same, regardless of layer sampled. Instead, we hypothesise that the target animal cells and therefore their DNA would be heterogeneously distributed throughout the faecal mass. Since faeces are unformed in the upper regions of the colon, exfoliated epithelial cells from those regions would likely be incorporated within the faecal mass through peristaltic contractions of the colon [13, 66].

The degree to which voided dugong faeces were exposed to the environment was found to significantly influence amplification success and relative amount of recovered DNA. Comparing mtDNA amplifications, highly eroded stools were found to amplify poorly compared to fresher ones. Eroded faeces were anticipated to amplify poorly as DNA degrades over time [67]. Epithelial cells undergo apoptosis as they detach from the basement membrane [68], where enzymes that degrade DNA break down the phosphodiester bonds that form the backbone of DNA [69]. This breaks the DNA into progressively shorter fragments and eventually into nucleotides [69]. The new HV-CTAB-PCI method, however, greatly improved mtDNA amplification, allowing consistent amplification even in eroded faecal samples. As faecal DNA is often highly degraded, the extraction of DNA from a larger amount of faecal material, used in the HV-CTAB-PCI method, increases the chance for more target DNA of sufficient length or quality to be extracted, thus allowing for higher amplification success. This newly developed DNA extraction method also enabled the comparison of ZFX amplifications in faeces of different environmental exposure levels (EELs). The amplification of ZFX marker was found to be highest in freshest faeces (*ex*-EE-L1) compared to older ones. Interestingly, the amplification success and relative quantity of mtDNA were found to be higher in fresh *in*-*ōceanum* faeces (*in*-EE-L3) compared to the *ex-ōceanum* faeces (*ex*-EE-L1), in contrast to what one might expect. The amount of amplifiable target DNA is influenced by the physical loss and chemical degradation (e.g., fragmentation) of DNA. Although the loss of target DNA could be higher when faeces is transported in the ocean, sea salts such as Ca^2+^ and Mg^2+^, for which DNA has a higher affinity compared to Na^+^ [70, 71], may help to stabilise or preserve the integrity of DNA [71–73]. Therefore, the higher relative quantity of mtDNA in *in*-EE-L3 faeces may be due to preservative effects of salt. However, the higher DNA loss in *in*-EE-L4 and L5 faeces may have outweighed any preservative effects on DNA, leading to the observed results. Although it remains unknown why the ZFX amplifications failed to reproduce the same trend, it is possible that structural differences (circular versus linear) or cellular location of mtDNA versus nDNA may have influenced their vulnerability to degradation [74].

Although this study achieved a low amplification success of nuclear markers (ZFX and SNPs) in more eroded faeces, the chance of success may be enhanced by scaling up the amount of starting faecal material used for DNA extraction, which can be performed easily using the HV-CTAB-PCI method without increasing the number of extractions. If that fails, separation of host DNA from bacterial DNA could be trialled using the host-DNA enrichment method by Chiou and Bergey [75] as this may improve the purity of DNA extracted. However, whole genome amplification may need to be performed prior to enrichment if the amount of target DNA extracted is insufficient for an efficient separation. Alternatively, host epithelial cells could be separated from a large proportion or the entire faecal mass using the methodology developed by Matsushita *et al*. [76], and DNA extraction could then be directly performed on those cells.

### SNP amplifications using faecal DNA

This study provides a proof-of-concept for the potential utilisation of faecal DNA in population genetic studies of dugongs, as all ten SNPs were successfully amplified and sequenced, using the dugong faecal DNA extracted with the new HV-CTAB-PCI method. The SNP primers did not significantly influence amplification success, but interestingly, primers Dug12 and Dug63 amplified less than other SNP primers. One plausible explanation is that those target sites may be more sensitive to DNA degradation. For example, Johnston *et al*. [77] showed that regions where SNP markers showed polyploidy in Atlantic salmon, due to historical genome duplication, had higher sensitivity to DNA degradation and thus lower genotyping success. Alternatively, the primers used in this study that were designed for those target sites may not have been optimal. The impact of DNA degradation was also reflected in SNP amplifications as fresh faeces amplified more successfully than eroded ones. Since SNP amplicons are generally shorter than microsatellites [32, 78], the difficulty for SNP amplification in eroded faeces may indicate that the DNA extracted from these faeces was too degraded or fragmented for primers to bind and amplify at the target site. That said, only one SNP marker (Dug54) was used to compare the amplification success between faeces of different EELs which limits the extent of inferences that can be made.

## Conclusions

This research has successfully pioneered a DNA extraction protocol (HV-CTAB-PCI) for an herbivorous marine mammal, the dugong, and has demonstrated the most efficient sampling methodologies to maximise retrieval of target DNA from faecal samples. These advances have enabled amplification of both mitochondrial and nuclear markers used in genetic population studies of dugongs. This study also provided an indication of the likely distribution of sloughed epithelial cells and thus target DNA within faecal stools, which may help researchers increase the efficiency and success for recovery of target DNA. Although it may be considered a limitation to not compare relative amounts of nDNA between outer and inner layers of faeces, the reliability and consistency in the amplification of mitochondrial markers rendered its use a more robust approach to ascertain where dugong DNA can be found in a faecal sample. The primary limitation of this research was the inability to isolate and amplify target nDNA of sufficient quality from the oldest, most eroded stools. Despite that, we have made multiple suggestions to increase the chances for DNA retrieval. Other practices that may influence the yield of target DNA, such as storage and preservation methods were not explored here as most samples were archived faeces collected years before the intent of the current study, but such comparisons have been made by others in previous work, e.g., [79–81].

This is the first study to demonstrate consistent amplification of nuclear markers (ZFX and SNPs) from dugong faeces, and the development of this HV-CTAB-PCI method will facilitate non-invasive genetic studies in areas where direct sampling is unfeasible. For instance, faecal samples have been collected from some remote and turbid water regions in northern Queensland where dugongs could not be sampled directly. These samples may now be analysed to gain a fuller understanding on the population structure of dugongs along the entire eastern Queensland coast. Future application of this approach could include broad-scale population genetic studies of dugongs throughout the Indo-Pacific region where resources for direct sampling are typically unavailable, but obtaining samples for genetic analysis is critical.

## Supporting information

S1 Fig Results from a pilot study conducted to determine initial amount of total DNA (10 and 100 ng) to be added into qPCR assay for comparison of mtDNA amplification between outer surface and inner core of faeces with different environmental exposure levels. All DNA was extracted using the QIAamp method. (A) Stacked bar charts of PCR success for the different initial total DNA used. No difference was found between the initial DNA amount used (χ^2^ = 0.381, p = 1.000). (B) Violin plots incorporating box plots of triplicate success for the different initial total DNA. No difference was found between the total DNA used (Kruskal-Wallis χ^2^ = 0.804, df = 1, p = 0.370).
(TIF)

S2 Fig Alignment of SNP sequences amplified using ten dugong SNP primers with two forms of alleles known. Three random samples were chosen to be shown in the plot for each primer set. (A) Primer Dug12. (B) Primer Dug40. (C) Primer Dug50. (D) Primer Dug51. (E) Primer Dug53. (F) Primer Dug54. (G) Primer Dug60. (H) Primer Dug62. (I) Primer Dug63. (J) Primer Dug64.
(TIF)

S1 File HV-CTAB-PCI DNA extraction protocol. (DOCX)

S2 File QIAamp DNA extraction protocol. (DOCX)
