# Supplementary figures and images for: A new DNA extraction method (HV-CTAB-PCI) for amplification of nuclear markers from open ocean-retrieved faeces of an herbivorous marine mammal, the dugong
Source: PLoS One. 2023 Jun 7;18(6):e0278792. doi: 10.1371/journal.pone.0278792 (PMC10246842; doi:10.1371/journal.pone.0278792)

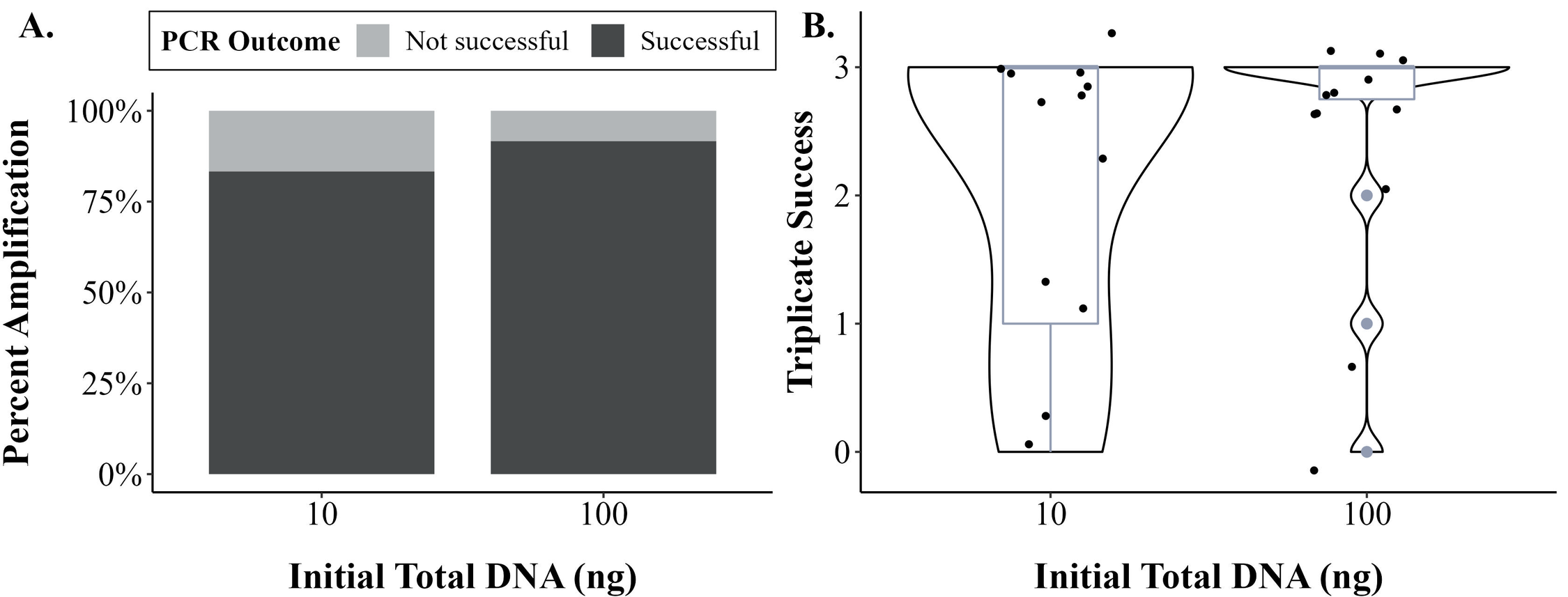

Supplement: S1 Fig — All DNA was extracted using the QIAamp method. (A) Stacked bar charts of PCR success for the different initial total DNA used. No difference was found between the initial DNA amount used (χ2 = 0.381, p = 1.000). (B) Violin plots incorporating box plots of triplicate success for the different initial total DNA. No difference was found between the total DNA used (Kruskal-Wallis χ2 = 0.804, df = 1, p = 0.370). (TIF) [file pone.0278792.s001.tif]

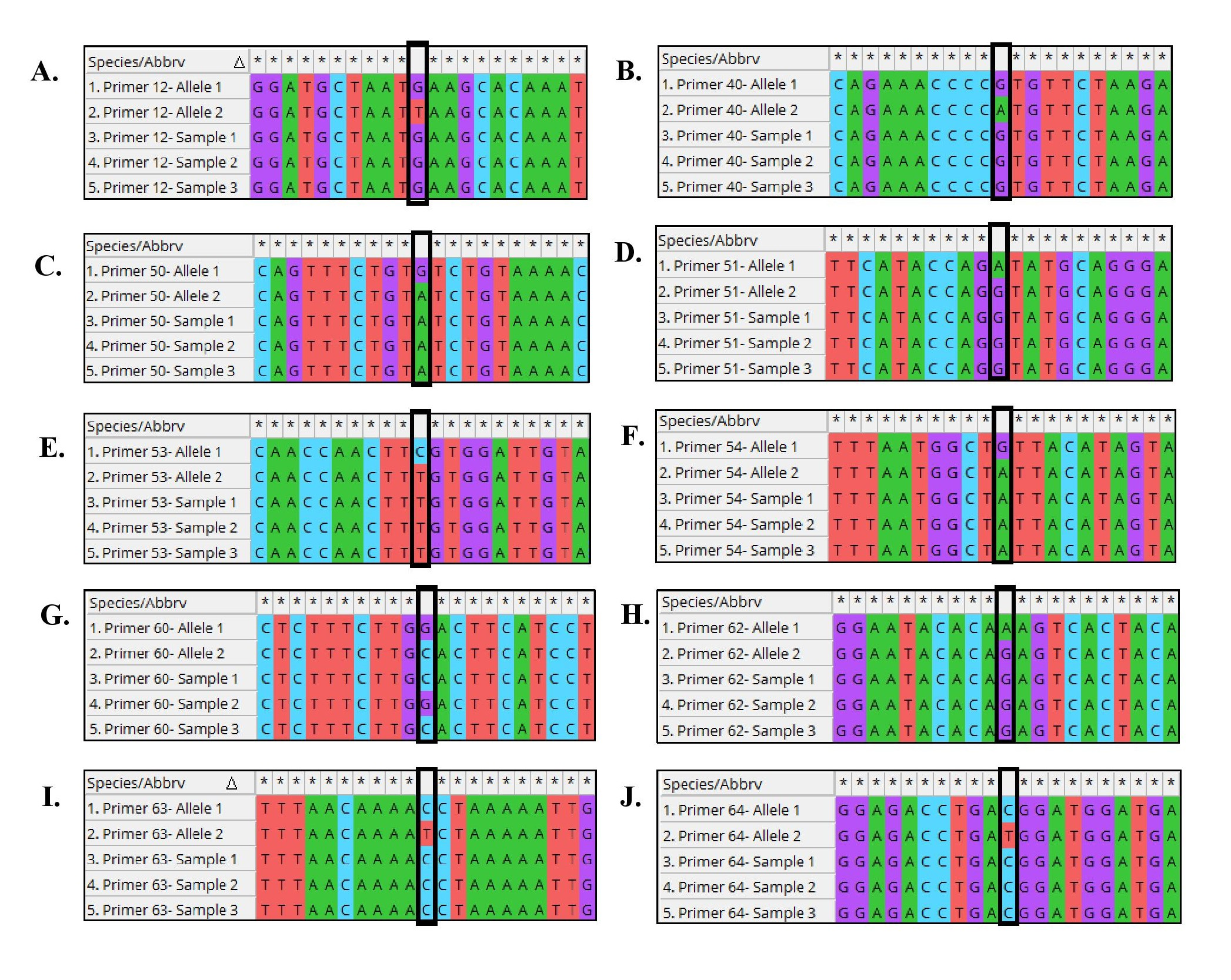

Supplement: S2 Fig — Three random samples were chosen to be shown in the plot for each primer set. (A) Primer Dug12. (B) Primer Dug40. (C) Primer Dug50. (D) Primer Dug51. (E) Primer Dug53. (F) Primer Dug54. (G) Primer Dug60. (H) Primer Dug62. (I) Primer Dug63. (J) Primer Dug64. (TIF) [file pone.0278792.s002.tif]
