# Supplementary material for: A new DNA extraction method (HV-CTAB-PCI) for amplification of nuclear markers from open ocean-retrieved faeces of an herbivorous marine mammal, the dugong
Source: PLoS One. 2023 Jun 7;18(6):e0278792. doi: 10.1371/journal.pone.0278792 (PMC10246842; doi:10.1371/journal.pone.0278792)
Supplement: S1 File — (DOCX) [file pone.0278792.s003.docx]

**HV-CTAB-PCI DNA Extraction Protocol**

1. Clean the working bench with TriGene disinfectant.
2. Scrape 1 g of faecal material from the outer surface of a faeces and put it into a 15 mL centrifuge tube.
3. Transfer the faecal material into a mortar and grind the faeces into powder with liquid nitrogen.
4. Add 1 mL of Lysis Buffer 1 (LB1: CTAB 2 %, Tris– HCL 100 mM, EDTA 20 mM, NaCl 1.4 M, pH 7.5) to the mortar containing the faecal material to further grind and mix in the buffer with the ground faeces. Transfer the liquid back into the 15 mL tube.
5. Add another 1 mL of LB1 to the mortar to mix in any leftover faecal material on the mortar and transfer the liquid back into the 15 mL tube. Repeat this step once more, and then add 2 mL of LB1 to the 15 mL tube containing the faecal homogenate. Thus, a total of 5 mL of LB1 would be added to the ground faeces.
6. Vortex the faecal homogenate and incubate in a thermomixer for 3 h, with occasional mixing, at 60°C for cell lysis.
7. Centrifuge the sample at 3,150 *g* (4,000 rpm) for 12 min and pipette 4 mL of the supernatant into a new 15 mL tube.
8. Add 4 mL of phenol: chloroform: isoamyl alcohol (21:20:1) to the supernatant, then gently mix the tube. Centrifuge the sample for 3 min at 3,150 *g* (4,000 rpm) and pipette 3 mL of the aqueous phase into a new 15 mL tube.
9. Add 330 µL of Lysis Buffer 2 (LB2: CTAB 10 %, NaCl 0.5 M, pH 5.5) to the aqueous phase, and leave it to lyse further at 60°C for 4 h.
10. Add 104 µL of protease to the sample and leave it to digest proteins at 60°C for another 1 h.
11. Add 3434 µL of phenol: chloroform: isoamyl alcohol (21:20:1) to the mixture, and then gently mix the tube well, and centrifuge the sample for 12 min at 3,150 *g* (4,000 rpm). Then, pipette 3 mL of the aqueous phase into a new 15 mL tube.
12. Add one volume of isopropanol (i.e., 3 mL) to precipitate the DNA overnight at -20°C.
13. Centrifuge the sample for 20 min at 8000 *g* (5,200 rpm), and then get rid of all the supernatant.
14. Add 400 µL of 70 % ethanol to the pellet to wash it. Vortex and then centrifuge the sample at 3,150 *g* for 12 min and get rid of the supernatant.
15. Dry the pellet in a fume hood at room temperature for 15 min.
16. Resuspend the pellet in 250 µL of TE buffer (10 mM Tris–HCl, 1 mM EDTA, pH 8).
17. Store the DNA isolate at -20°C for use within a week or at -80°C for longer-time storage.
