# Supplementary material for: A new DNA extraction method (HV-CTAB-PCI) for amplification of nuclear markers from open ocean-retrieved faeces of an herbivorous marine mammal, the dugong
Source: PLoS One. 2023 Jun 7;18(6):e0278792. doi: 10.1371/journal.pone.0278792 (PMC10246842; doi:10.1371/journal.pone.0278792)
Supplement: S2 File — (DOCX) [file pone.0278792.s004.docx]

**QIAamp DNA Extraction Protocol**

1. Use a sterile blade to scrape off 220 mg of faecal material from the outside surface of a stool and then transfer it into a 2 mL microcentrifuge tube.
2. Transfer the faecal material into a mortar and grind the faeces into powder with liquid nitrogen.
3. Add 500 μL of InhibitEX buffer to the mortar containing the faecal material to further grind and mix in the buffer with the ground faeces. Transfer the liquid back into the 2 mL tube.
4. Add another 500 μL of InhibitEX buffer to the mortar to mix in any leftover faecal material on the mortar. Transfer the liquid back into the 2 mL tube.
5. Vortex continuously for 1 min or until the solid material is thoroughly homogenised.
6. Centrifuge the sample at 20,000 *g* (~14,000 rpm) for 2 min to pellet stool particle.
7. Pipette 25 μL of Proteinase K into a new 2 mL tube.
8. Pipette 800 μL of supernatant from the centrifuged homogenate into the 2 mL microcentrifuge tube containing Proteinase K.
9. Add 800 μL of Buffer AL to the mixture and vortex for 15 s.
10. Incubate at 70°C for 10 min. Then, centrifuge briefly to remove drops from the inside of the tube lid.
11. Split the lysate into two 2 mL tubes (~813 μL each tube).
12. Add 400 µL of 99.9 % ethanol to both tubes containing the lysate (thus, 800 µL of ethanol overall) and mix by vortexing.
13. Carefully apply 600 μL of lysate to the QIAamp spin column. Close the cap and centrifuge at 20,000 *g* (~14,000 rpm) for 1 min. Place the QIAamp spin column in a new 2 mL collection tube and discard the tube containing the filtrate.
14. Repeat step 13 until all the lysate has been loaded on the column.
15. Carefully open the QIAamp spin column and add 500 µL of Buffer AW1. Centrifuge at 20,000 *g* (~14,000 rpm) for 1 min. Place the QIAamp spin column in a new 2 mL collection tube and discard the collection tube containing the filtrate.
16. Carefully open the QIAamp spin column and add 500 µL of Buffer AW2. Centrifuge at 20,000 *g* (~14,000 rpm) for 3 min. Place the QIAamp spin column in a new 2 mL collection tube and discard the collection tube containing the filtrate.
17. Centrifuge at 20,000 *g* (~14,000 rpm) for 3 min to eliminate the chance of possible Buffer AW2 carryover.
18. Transfer the QIAamp spin column into a new, labelled 1.5 mL microcentrifuge tube and pipette 100 µL of Buffer ATE directly onto the QIAamp membrane to elute the DNA from the spin column into the 1.5 mL Eppendorf LoBind microcentrifuge tube. Incubate for 1 min at room temperature, then centrifuge at 20,000 *g* (~14,000 rpm) for 1 min to elute DNA.
19. Store the DNA isolate at -20°C for use within a week or at -80°C for longer-time storage.
